# Disruption of ecological networks in lakes by climate change and nutrient fluctuations

**DOI:** 10.1038/s41558-023-01615-6

**Published:** 2023-03-23

**Authors:** Ewa Merz, Erik Saberski, Luis J. Gilarranz, Peter D. F. Isles, George Sugihara, Christine Berger, Francesco Pomati

**Affiliations:** 1 Department of Aquatic Ecology, Eawag: Swiss Federal Institute of Aquatic Science and Technology, Dübendorf, Switzerland; 2 Scripps Institution of Oceanography, University of California San Diego, La Jolla, CA USA; 3 Vermont Department of Environmental Conservation, Montpelier, VT USA; 4 Stadt Zuerich, Wasserversorgung, Qualitaetsueberwachung, Zuerich, Switzerland

**Keywords:** Ecological networks, Climate-change ecology, Freshwater ecology, Food webs

## Abstract

Climate change interacts with local processes to threaten biodiversity by disrupting the complex network of ecological interactions. While changes in network interactions drastically affect ecosystems, how ecological networks respond to climate change, in particular warming and nutrient supply fluctuations, is largely unknown. Here, using an equation-free modelling approach on monthly plankton community data in ten Swiss lakes, we show that the number and strength of plankton community interactions fluctuate and respond nonlinearly to water temperature and phosphorus. While lakes show system-specific responses, warming generally reduces network interactions, particularly under high phosphate levels. This network reorganization shifts trophic control of food webs, leading to consumers being controlled by resources. Small grazers and cyanobacteria emerge as sensitive indicators of changes in plankton networks. By exposing the outcomes of a complex interplay between environmental drivers, our results provide tools for studying and advancing our understanding of how climate change impacts entire ecological communities.

## Abstract (web summary)

The authors investigate the impact of warming and nutrient supply on entire ecological networks within ten Swiss lakes. Warming generally reduces network interactions, particularly under high phosphate, leading to shifts in trophic control of food webs.

## Main

Human impacts, such as climate change and pollution, are reorganizing entire ecosystems by affecting the nature and strength of ecological interactions and, thereby, the composition of natural communities^1–4^. Ecological interactions between species are the engine of community dynamics and ecosystem processes, although they remain perhaps the most overlooked component of biodiversity change^5,6^. Studying the structure and dynamics of interactions in a community, which can be conceptualized as information networks, has proved to be fundamental to understand how global change alters ecosystem structure and function^7^. Although it is known that human activities affect ecological networks, researchers and stakeholders urgently need tools to understand and predict the contemporary and non-additive effects of different stressors^2,8–10^. The warming experienced by many lakes, particularly in the past decade, has put ecological networks in a situation where slight increases in nutrient levels can trigger dramatic ecosystem changes^11–13^.

Changes in network properties can portend the possibility of rapid shifts in community structure and increase species extinction risks^12–15^. Networks vary over space and time in the number of interactions between taxa (that is, addition or loss of connections) or the strength of interactions (for example, rerouting of biomass flows through existing connections)^2,16^. Network connectance and the strength of species interactions—particularly in trophic networks—are structural properties that can signal large-scale changes in the whole ecosystem, with potential implications for ecosystem stability (in the sense of Lyapunov stability of equilibria) and the maintenance of biodiversity^2,16,17^.

Knowledge about how entire interaction networks reorganize as a consequence of global change is, however, limited owing to many challenges, including the scarcity of long-term, well-curated time series of complete ecological networks^18^, which, when available, are often characterized by complex nonlinear interactions and require specific inference methods^19,20^. Most research also focuses on only one type of interaction (for example, trophic, mutualistic or competitive). Until recently, ecological theory and practice have often assumed that interactions are fixed and constant over time^19,21^. Moreover, it has been difficult to assess how the properties of ecological networks respond to interacting environmental stressors. This is because of the limited available empirical data on large-scale ecosystems and the need for quantitative data-analytic methods to address complex relationships that vary with system state. Previous work on ecological networks, mostly theoretical and based on many assumptions, offers only partial expectations of how natural ecosystems may respond to multiple anthropogenic stressors in reorganizing taxa interactions^22,23^.

**Fig. 1:**
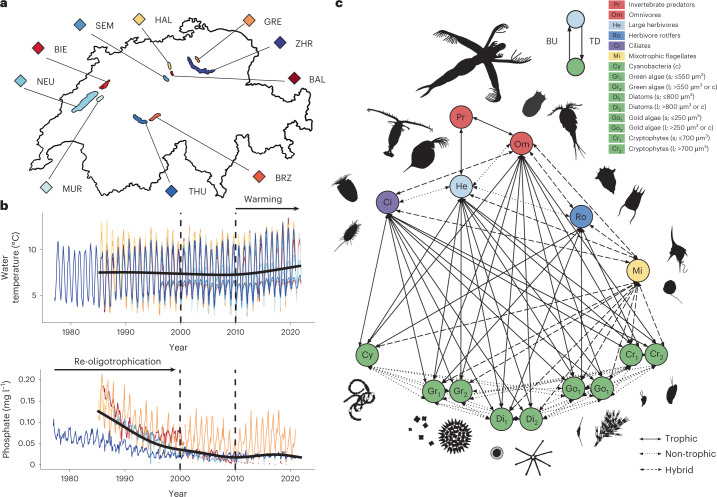
Environmental change in ten Swiss lakes over the past five decades and its implications for plankton networks. **a**, Study sites belong to the same geographic region. Size and distance are to scale. **b**, Monthly phosphate concentrations (PO_4_) and water temperature averaged over the water column. Colours represent single lakes; black line is a smoothing average across lakes; dashed vertical lines indicate periods in the lakes’ histories (end of re-oligotrophication—circa year 2000, and increase in net lake warming—circa 2010). **c**, Conceptual model of a plankton network in temperate lakes. s, small single cell; l, large single cell; c, colonial (Methods). Non-trophic links encompass facilitation and competition. Trophic links represent predator–prey interactions. Hybrid links can be both trophic and non-trophic; for example, mixotrophic protists can prey on and/or compete with other phytoplankton species. Hybrid and trophic links go from the bottom to the top of the food web, that is, from a primary producer to a grazer (bottom-up, BU) or from top to bottom, that is, from a grazer to a primary producer (top-down, TD).

**Extended Data Fig. 1:**
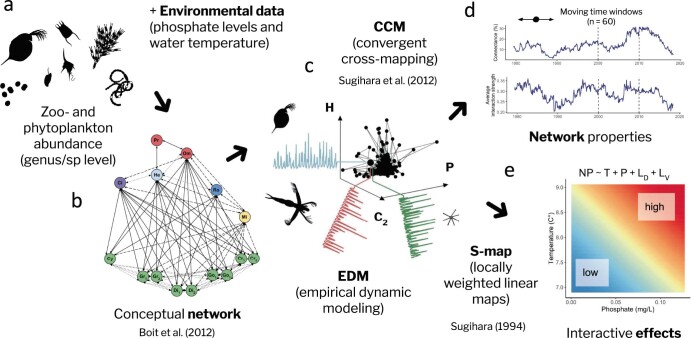
Methods summary. **[a]** We used zoo- and phytoplankton abundance data (at the genus or species level) from 10 Swiss lakes in the peri-alpine region, along with environmental data (phosphate concentration and water temperature). **[b]** Phyto- and zooplankton abundance data were sorted into a conceptual network, representative for planktonic ecosystems in temperate lakes. **[c]** We used a non-parametric approach called empirical dynamic modeling (EDM), in particular, a non-linear causality test referred to as convergent cross mapping (CCM), **[d]** to estimate changes in network properties (number of realized links and strength of those links) over time (within a 60-month moving window). **[e]** To study how network properties respond to warming and re-oligotrophication, we modeled the time series of network properties with S-maps (also part of the EDM framework) using temperature (T), phosphate concentration (P) and a lake’s depth (L_D_) and volume (L_v_). These models predict network properties over a gradient of phosphate (x-axis) and water temperature (y-axis), ranging from the minimum to the maximum values observed across all lakes. This allowed us to explore the non-additive effect of water temperature and phosphate availability on network properties.

Here, we address these gaps by studying the effects of two major anthropogenic stressors on plankton networks: warming and nutrient pollution^12,24^. Specifically, we examined re-oligotrophication, referring to the process of controlled phosphorus reduction to revert lakes to their original state before anthropogenic nutrient pollution. We measure the temporal changes in connectance and interaction strengths at three levels: (1) the whole network; (2) top-down and bottom-up links that control food-web dynamics; and (3) different interaction types. To understand the interdependent effects of warming and oligotrophication on plankton networks, we examined 20–43 years of well-curated monthly plankton community data across ten peri-alpine Swiss lakes (Fig. 1a, Supplementary Table 1 and Extended Data Fig. 1a). Data from five lakes cover more extensively the process of re-oligotrophication, and eight the period of net warming (Methods). This is a long-term and consistent historical series of an entire ecological network along with measurements of abiotic environmental variables, which is very rare in ecology. Thus, these data provide a timely opportunity to investigate network-wide consequences of climate change and nutrient fluctuations in natural lake ecosystems.

We first analyse the data collected from the ten Swiss lakes for the phosphate levels and temperature trends. Dissolved inorganic phosphorus (phosphate) is the main limiting factor for phytoplankton growth in temperate lakes and the principal driver of eutrophication^24^. Temperature is a fundamental driver of metabolic responses in plankton, influencing competitive and trophic dynamics and, consequently, how biomass is partitioned and distributed within an ecosystem^11,25^. Starting in the 1970s, the lakes considered in this study underwent managed re-oligotrophication to control the release of phosphorus into the ecosystems (Fig. 1b). At the same time, the average water-column temperature has steadily increased since the 1950s^26^.

**Extended Data Fig. 2:**
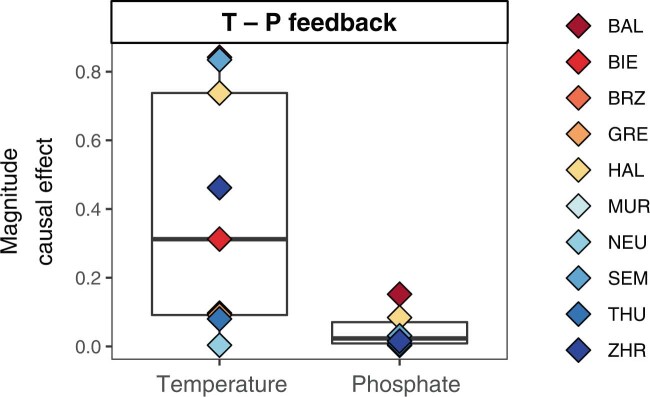
Water temperature influences phosphate levels but not vice versa. The magnitude of the effect was calculated by multiplying the predictive skill (rho) by the prevalence of the focal causal link over time and was corrected for seasonality (Methods). Diamonds represent averages over time within lakes (only lakes with significant effects are displayed.); boxes’ lower and upper hinges (bounds) correspond to the first and third quantiles (the 25th and 75th percentiles) across all lakes; while black bars represent the median (50th percentile). The upper whisker (max) extends from the hinge to the largest value no further than 1.5 the distance between the first and third quartile. The lower whisker (min) extends from the hinge to the smallest value at most 1.5 the distance between the first and third quartile of the hinge. Data beyond the end of the whiskers are outliers.

Warming can also indirectly affect nutrient supply in deep and temperate lakes such as the ones studied here through changes in stratification^25,27^. A temperature rise reduces turbulence and deep mixing, decreasing the resuspension of phosphorus from the deep and nutrient-rich waters^26,28,29^. From 2010 to 2020, the average water-column temperature in the studied lakes rose by 0.4 °C to 1.7 °C (Fig. 1b and Supplementary Table 1), similar to the increase observed over the previous 60 years (1950–2010)^26,30^. To analyse the relationship between phosphate levels and water temperature in our datasets, we use a nonlinear causality test (convergent cross-mapping, CCM) from the empirical dynamic modelling (EDM) framework (Methods). From this, we find that water-column temperature causally influences changes in phosphate levels (a negative effect is expected^25,28,29^), but not vice versa (Extended Data Fig. 2). This unidirectional relationship suggests that warming, by regulating phosphate availability, may have a more pervasive influence on plankton networks than would be expected by the effects of water temperature alone.

**Extended Data Fig. 3:**
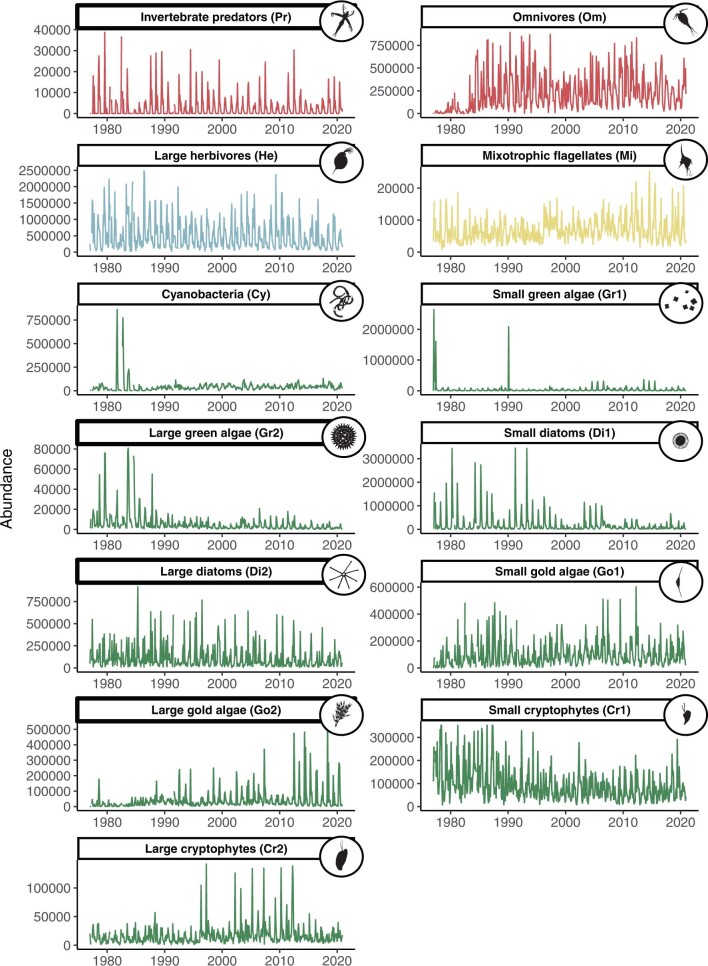
Time series of plankton guilds (nodes in the network) in Lake Zurich. Abundances of He-Pr are in indv./m2 while Ro-Mi and Cy-Cr2 are in indv./L. He-Pr has been collected over the whole water column, whereas Ro-Mi and Cy-Cr2 were sampled in the photosynthetic zone (Supplementary Table S1). Lake ZHR did not consider small grazers (Ro, Ci) during the sampling. Other lakes are displayed in Supplementary Fig. S1.

To study ecological networks, which are expected to be affected by warming^3,31^, we group the plankton species present in the lakes into well-known trophic guilds^32,46^ based on species’ body size, nutrition requirements and foraging behaviour (Extended Data Fig. 1b). The resulting conceptual network consists of up to 15 nodes (Fig. 1c and Methods) comprising the following: invertebrate predators, omnivores, large and small herbivores, mixotrophic flagellates, and primary producers. Guilds of primary producers (phytoplankton) represent the base of aquatic food webs and, worldwide, they account for roughly half of the global primary production^33^. When possible, we divided each of their guilds into two nodes based on cell size and coloniality (Fig. 1c). Each node in the network contains a time series of monthly abundances that records how guilds wax and wane over time, while the number of nodes per lake remains constant (Extended Data Fig. 3, and Supplementary Figs. 1 and 2). These time series contain essential information about how guilds influence each other and themselves (that is, dynamic links). We consider direct (for example, predator–prey) and indirect links (for example, competition for resources) as taxa interactions.

To employ this established network for studying how interactions change as a function of system state (as reasonably expected from nonlinear systems^19,34^), we use CCM to identify causal associations between network nodes and quantify their strength (that is, cross-map accuracy; Extended Data Fig. 1c)^34^. CCM quantifies how changes in one time series (the driven variable, predictor) can predict changes in another (the causal driver, predictee, that is, how much information about the driver is contained in the driven variable). To obtain the causal relationships between guilds, we minimize the intra-annual signal of the environmental drivers. Interactions are corrected for seasonality by assuming no interaction when the interaction strength given by CCM is lower than that of a seasonal surrogate null model^35,36^ (Methods). By measuring cross-map accuracy (Pearson’s correlation between predictions and observations—rho) in a 60-month moving window, we track how causal associations between network nodes and their strength vary in each lake network. Thus, within each window, we measure the following: (1) connectance as the percentage of significant associations between nodes (causal links), *C* = 100 × (*L*/*N*(*N*−1)), where *L* is the number of interactions between nodes and *N* is the number of nodes in the system; and (2) interaction strengths among causal links (cross-map accuracy; Extended Data Fig. 1d). The window size was chosen to encompass more than a year and avoid strong seasonal signals, while not being too long to miss key trends^30^. The main trends in connectance and interaction strength reported below are robust to the choice of window size (Supplementary Fig. 3).

**Fig. 2:**
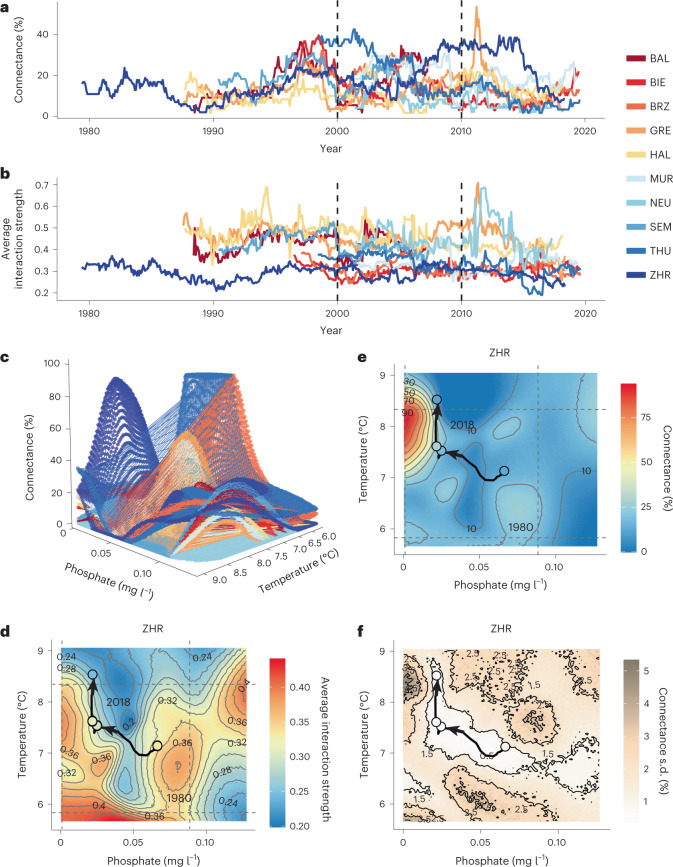
Connectance and interaction strength between nodes of plankton networks are dynamic over space and time, and exhibit nonlinear relationships with water temperature and phosphorus. **a**,**b**, Connectance (percent realized links, **a**; Fig. 1c) and average strength between realized links (**b**); time series are based in the centre of a moving window of 60 months used for causality detection via CCM. **c**–**f**, S-map models’ inferred interactions between average water-column phosphate and temperature on realized network properties. **c**, Example three-dimensional plot depicting the interactive effects of water temperature and phosphate on all lakes, which emerge from predicting network properties (*z* axis) over varying levels of the chosen pair of explanatory variables while keeping lake depth and volume constant (Methods). **d**, Colour-coded contour plots of predicted strength of network links in Lake ZHR. Dots represent the start/end of the re-oligotrophication and net-warming periods; trajectories show the direction of time, with an arrowhead pointing to the end of each period. The displayed years are the middle point of a five-year time window. Dashed lines show 95% ranges of empirical input values of temperature and phosphate. **e**, Colour-coded contour plots of predicted network connectance in Lake ZHR; lines and dots as in **d**. **f**, Standard deviation of predicted connectance, estimated over 100 model predictions using 50% of the data.

**Fig. 3:**
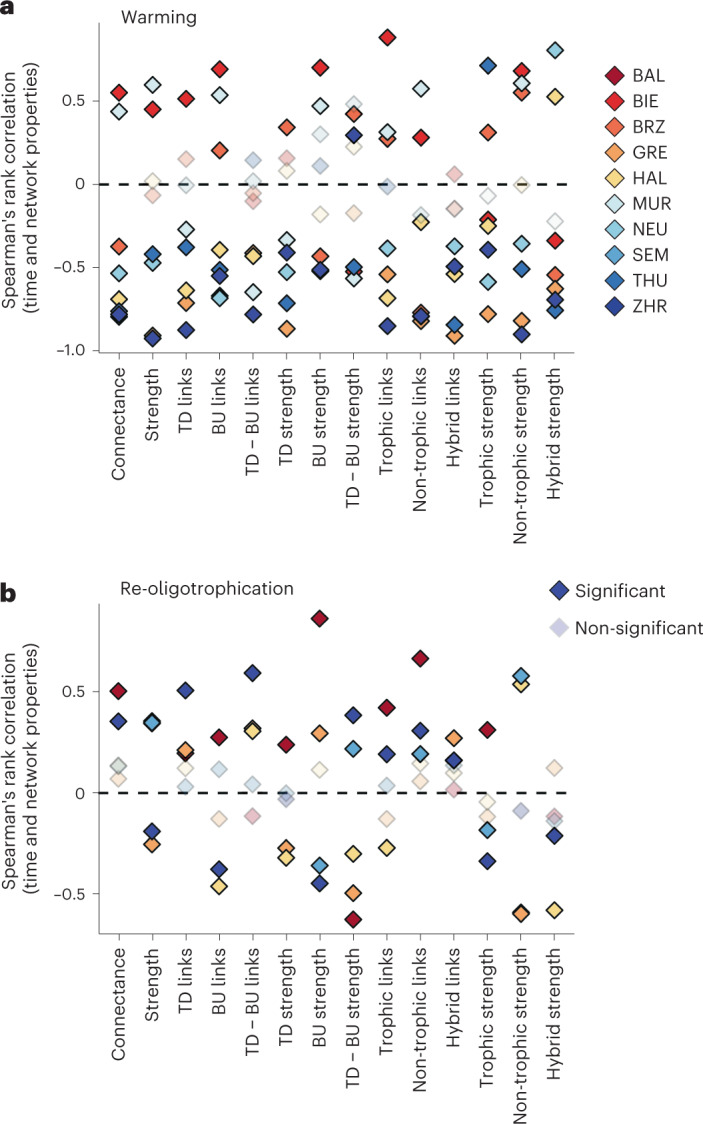
Lake warming leads to a generalized decrease in network connectance and interaction strength. **a**,**b**, Spearman’s rank correlation between network properties and time, during the two focal periods of lakes’ history. To study correlations during warming (**a**), we used data points from 2000 up to the present (BIE, BRZ, GRE, HAL, MUR, NEU, THU and ZHR), while data from before the year 2000 (lakes BAL, GRE, HAL, SEM and ZHR) were used to study lake re-oligotrophication (**b**).

At the network level, connectance and average interaction strengths vary over time in each lake (Fig. 2a,b). The observed time series shows how network connectance increases significantly in two out of five lakes during re-oligotrophication (for example, +4.2% in Lake Zurich, Spearman’s rank correlation *R* = 0.35, *P* < 0.001; Figs. 2c and 3b, and Methods). Connectance decreases significantly in six out of eight lakes when warming accelerates (for example, 14.8.% in Lake Zurich, *R* = −0.78, *P* < 0.001; Figs. 2a and 3a). Those trends agree with recent evidence from natural freshwater systems^3^ that warming reduces the connectance of ecological networks. The average interaction strength among plankton guilds is less variable over time than network connectance and exhibits lake-specific trends during re-oligotrophication and warming (Figs. 2b and 3). The observed trends in connectance and interaction strength emerge from the interdependent effects of re-oligotrophication and warming (for example, Extended Data Fig. 2), and can depend on other internal lake factors; they therefore cannot be studied based on correlation alone.

To study the interdependent relationship between network properties and environmental factors, we model the effects of contrasting gradients of decreasing phosphorus and lake warming on the network structure in all the lakes in our dataset (Fig. 1b and Extended Data Fig. 1e). Using S-maps to account for time-varying relationships (Methods), we model network properties as a function of the interaction between temperature, phosphate, and the lake’s depth and volume^24,25^. These models allow us to disentangle the effect of warming, usually convolved with re-oligotrophication, across all lakes, while considering lake morphometric differences and idiosyncrasies (Fig. 2c). The different lakes can be used as independent case studies as they belong to the same geographic and climatic region, went through a similar history of re-oligotrophication and warming, and share the same plankton species pool. Moreover, within the observed temperature and phosphate levels ranges, we can now make predictions for previously unobserved temperature and phosphate level combinations across lakes. We averaged predictions over 100 models using 50% of the data to get an uncertainty estimation around model predictions (Fig. 2f).

We found that network connectance and interaction strength show highly nonlinear and lake-specific responses to changes in phosphate concentration and water temperature (Fig. 2c, Supplementary Table 2 and Supplementary Fig. 4). For most lakes, S-map models predict maximum connectance and interaction strength at intermediate water temperature and phosphorus (Supplementary Figs. 4 and 5). Raising water temperature is predicted to negatively affect connectance and interaction strength in most lakes, while increasing phosphorus levels result in lake-specific responses, which are strongly dependent on temperature (Supplementary Figs. 4 and 5). Our models also show higher uncertainty in predicting network properties for the combination of high temperature and phosphorus (Supplementary Fig. 5). As an illustrative example of these ecosystem responses, we display and discuss the predictions for Lake Zurich (Fig. 2d–f; see other lakes in Supplementary Fig. 5). In our dataset, Lake Zurich represents a median-sized, well-studied ecosystem and is economically important for fisheries and drinking water supply^37^. Moreover, this lake has the most consistent record (longest time series), encompassing re-oligotrophication and warming.

In Lake Zurich, the S-map models predict high connectance at medium water temperature and low phosphate levels, and low connectance at high temperature and medium phosphate levels (Fig. 2e). The historical trajectories indicate that Lake Zurich is now, after being warmed, in a state in which a slight increase in nutrients could drastically reduce network connectance. A further reduction in nutrients could highly promote connectance and increase interaction strength (Fig. 2d,e). This is expected because a reduction in nutrient levels can increase competition for resources and thus lead to stronger interactions^38,39^. For a given number of taxa, many strong interactions can negatively affect Lyapunov ecosystem stability, similar to a decrease in network connectance^16,17^. In a warming world, an ecosystem like Lake Zurich is now in a situation where a slight change in nutrient levels could have dramatic consequences for the whole network and thus ecosystem stability.

We next examined the directionality of trophic control. By using CCM, we calculate the relative frequency of causal effects descending (top-down links, for example, predator controls prey abundance) and ascending (bottom-up links, for example, prey controls predator abundance) through the network (Methods). It is known that warming can alter the metabolic rates of producers and consumers differently, influencing the strength of trophic interactions and the direction of trophic controls (for example, consumers controlling the population of their resource or vice versa), especially under reduced nutrient levels^3,11,40^. Experimental evidence suggests that warming strongly impacts aquatic food-web interactions by reducing trophic transfer efficiency^40–42^, and may also affect trophic controls in lakes, particularly when co-occurring with changes in nutrient availability^4,27,43^. If warming shifts control to bottom-up, where resources control consumers, the system dynamics become sensitive to nutrient inputs^11,44^. If top-down forces control a system, managing the lake’s productivity would require food-web manipulations (for example, stocking of piscivorous fish)^45^.

**Fig. 4:**
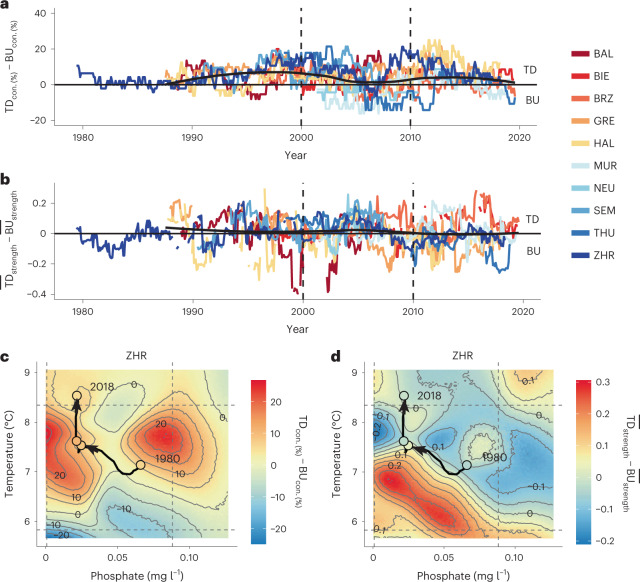
Warming reduces the number and strength of top-down relative to bottom-up network links, suggesting shifts in trophic controls. **a**,**b**, Difference between realized top-down and bottom-up links (**a**) and average strength of top-down and bottom-up links (**b**). Con., connectance. Positive values mean more or stronger top-down links; smoothing line shows trend of top-down versus bottom-up links based in the centre of a moving window of 60 months, used for CCM. **c**,**d**, Interactive effects of average water-column phosphate and temperature on realized top-down relative to bottom-up links (**c**), and their relative strength (**d**) in Lake ZHR. Colour-coded contour plots depict the S-map model inferred relationships as in Fig. 2 (Methods).

**Extended Data Fig. 4:**
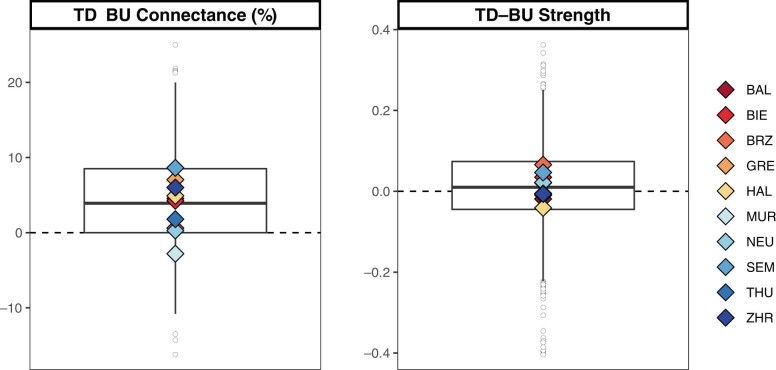
Trophic controls across lakes. The direction of controls across lakes, where positive values mean the system is mostly top-down controlled (predators control prey), and negative values mean the lake is mostly bottom-up controlled (predators are controlled by their prey). Points represent averages over time within lakes; boxes’ lower and upper hinges correspond to the first and third quantiles (the 25th and 75th percentiles) across all lakes; while black bars represent the median.

We find that top-down causal links are more frequent than bottom-up links in nine out of ten lakes (Fig. 4a and Extended Data Fig. 4), although, in half of the lakes, the bottom-up links are stronger on average (Fig. 4b and Extended Data Fig. 4). Trophic controls can covary, yet they may not do so with the same magnitude, and the difference between their number and strength fluctuates (Supplementary Fig. 6 and Fig. 4 a,b). Bottom-up and top-down controls are expected to change over time and across environmental gradients^4^. We find that in three lakes, re-oligotrophication slightly increased the number of top-down links relative to bottom-up (Figs. 4a and 3d). Warming in lakes, however, generally decreased the number of top-down links relative to bottom-up links (Figs. 4a and 3a).

To reveal the responses of bottom-up and top-down controls to phosphate concentration and warming, we use S-map models analogous to those used to predict connectance and interaction strength. S-map models predict that most lakes display a high prevalence and strength of top-down controls, relative to bottom-up, under low or intermediate water temperatures. At the same time, the effects of phosphorus depend on the specific lake (Supplementary Figs. 4 and 5). Models generally predict that under high water temperatures and high phosphate levels, plankton networks in lakes are bottom-up controlled (Supplementary Fig. 5). Taking Lake Zurich as an example, an increase in nutrient levels under the current climatic conditions would lead to plankton networks being strongly bottom-up controlled. Although combined warming and nutrient levels have system-specific and idiosyncratic shifts in bottom-up and top-down controls^11,44^ (Supplementary Fig. 4), our results suggest that under warming conditions, resources increasingly control consumers in planktonic food webs, and this could occur particularly when phosphorus levels are high. Moreover, S-map models display higher uncertainty for predicting the direction of trophic controls when phosphorus is high, reducing our ability to forecast future ecosystem states (Supplementary Fig. 5).

**Fig. 5:**
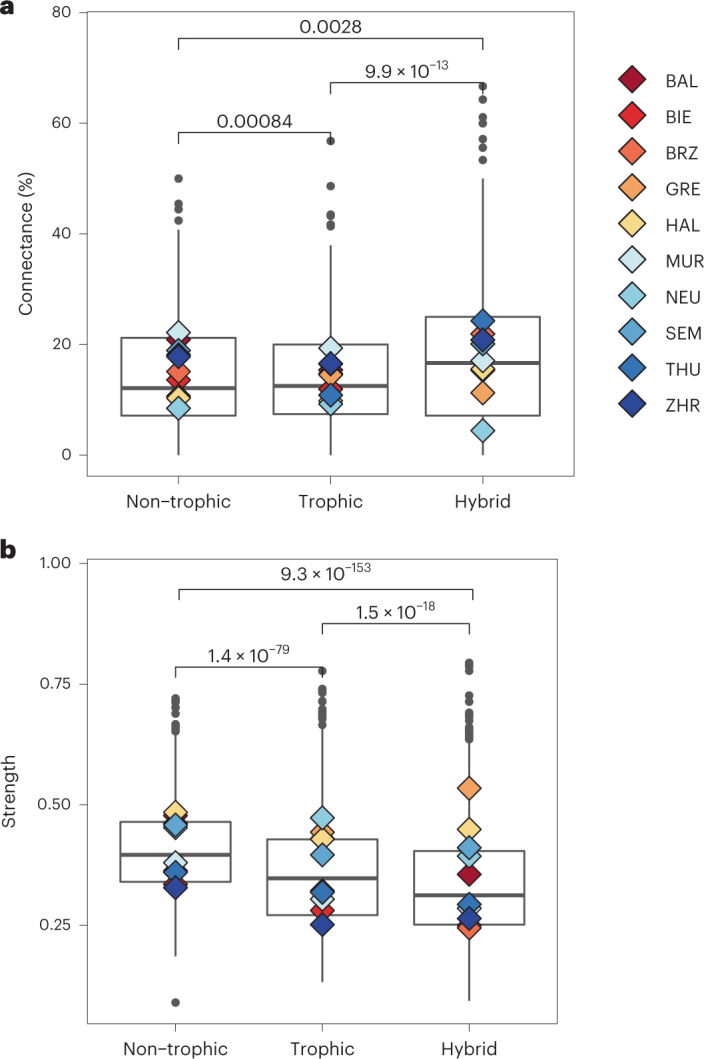
Hybrid links are the most common, whereas non-trophic interactions are the strongest. **a**,**b**, Prevalence (**a**) and strength (**b**) of trophic, non-trophic and hybrid links. Diamonds represent averages over time within lakes; boxes’ lower and upper hinges (bounds) correspond to the first and third quantiles (the 25th and 75th percentiles) across all lakes; and black bars represent the median (50th percentile). The upper whisker (maximum) extends from the hinge to the largest value no further than 1.5× the distance between the first and third quartile. The lower whisker (minimum) extends from the hinge to the smallest value at most 1.5× the distance between the first and third quartile of the hinge; data beyond the end of the whiskers are outliers. *P*-values are shown above the brackets and were calculated using pairwise comparisons and a Wilcoxon test (non-parametric).

So far, our results have shown how community-level responses—conceptualized by an ecological network—vary with temperature and phosphate concentration. To better understand the mechanisms leading to the observed network reorganization, we examine how different interaction types and guilds contribute to the temporal changes in connectance, interaction strength and trophic controls. We obtain, across all lakes, the frequency and average strength of trophic, non-trophic and hybrid links, the last of which can be both trophic and non-trophic depending on the conditions (for example, mixotrophic flagellates that change nutrition mode, or network associations involving large zooplankton that both prey on and compete with microzooplankton such as rotifers and ciliates). The temporal dynamics show that hybrid links are significantly more common in the causal network than non-trophic and trophic links (+~4.5% realized links; Fig. 5a and Supplementary Fig. 7). Non-trophic links were strongest on average in all lakes, while hybrid links were weakest (Fig. 5b and Supplementary Fig. 7). These results, showing that hybrid links account for the most connections while displaying weak interactions, support the hypothesis that intermediate consumers and generalists are important indicators of key structural changes in ecological networks^2^, underlying ecosystem stability^16,17^.

**Extended Data Fig. 5:**
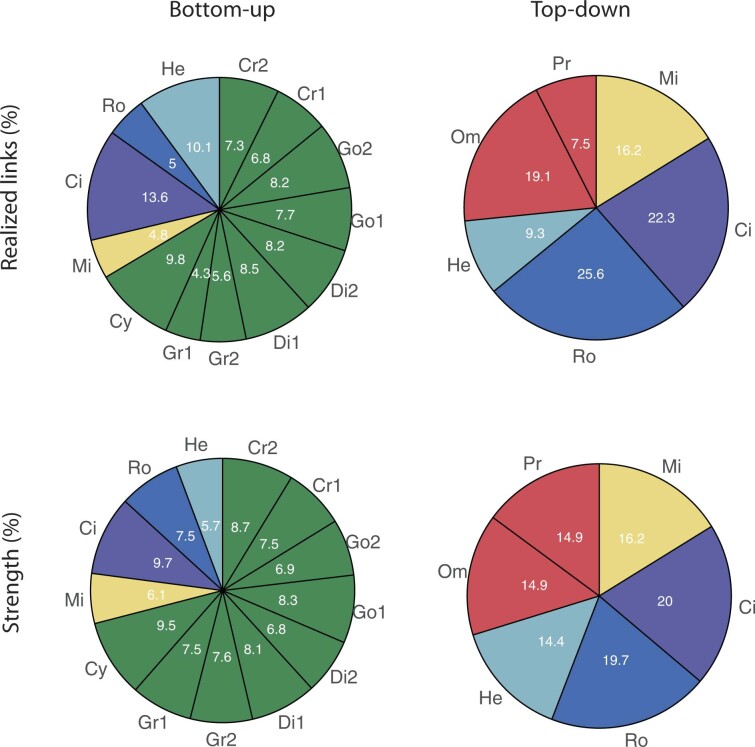
Relative contribution of plankton guilds to bottom-up and top-down network connectance (%) and strength. The direction of controls across lakes, where positive values mean the system is mostly top-down controlled (predators control prey), and negative values mean the lake is mostly bottom-up controlled (predators are controlled by their prey). Diamonds represent averages over time within lakes; boxes’ lower and upper hinges (bounds) correspond to the first and third quantiles (the 25th and 75th percentiles) across all lakes; while black bars represent the median (50th percentile). The upper whisker (max) extends from the hinge to the largest value no further than 1.5 the distance between the first and third quartile. The lower whisker (min) extends from the hinge to the smallest value at most 1.5 the distance between the first and third quartile of the hinge. Data beyond the end of the whiskers are outliers.

**Extended Data Fig. 6:**
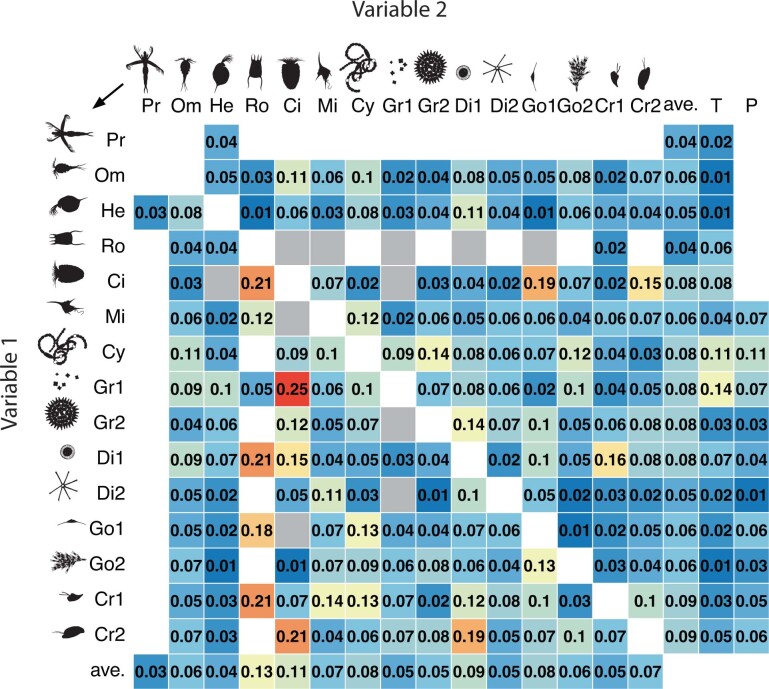
Interaction matrix between plankton guilds: ciliates are the most strongly connected guilds in the network. Interaction strengths were calculated by multiplying the average strength (rho, corrected for seasonality) by its prevalence (that is if a link were significant within 50% of time windows, prevalence would be 0.5). The direction of the interaction is Variable 2 → Variable 1. Ave. is the average interaction strength for each guild. T is water temperature and P is phosphate levels. White tiles are interactions not occurring based on the conceptual network (Fig. 1c) and grey tiles are interactions that were not significant in none of the lakes based on a convergence test in CCM.

By shifting our focus from interactions to the constituent nodes, we find that trophic controls are dominated, both in terms of realized links and interaction strengths, by small grazers (that is, rotifers, ciliates and mixotrophic flagellates; guilds Ro, Ci and Mi, respectively) and colonial cyanobacteria (Cy; Extended Data Fig. 5), and these guilds are broadly connected in the network (Extended Data Fig. 6). The abundances of ciliates and cyanobacteria are also strongly influenced by long-term changes in temperature (Extended Data Fig. 6). These findings agree with previous knowledge of small grazers being more resilient to environmental change due to their plastic nutrition strategies and foraging behaviour. As a central regulator of plankton food webs^32,46^, they influence community structure^25^ and food-web dynamics^2^, and it would be essential to include them in future monitoring programmes. Colonial, bloom-forming (and often toxic) cyanobacteria, which are an important issue for water quality, are, as expected, strongly driven by changes in phosphate levels and temperature^47^, but also broadly connected in the network, suggesting pervasive effects of this guild on plankton taxa interactions (Extended Data Fig. 6).

Our results provide much-needed information about how lake plankton interaction networks, paramount for the functioning of aquatic ecosystems, respond to climate change and nutrient pollution. We show that, while a reduction in phosphorus levels in most lakes increases network connectance, lake warming overall leads to a decrease in connectance and interaction strength (Fig. 3). We acknowledge that changes in the abundance or behaviour of important keystone taxa like fish or bivalves could have influenced the observed network dynamics. While we are missing continuous, reliable and unbiased estimates for both guilds in our study lakes, our approach carries information on all the interacting taxa and abiotic drivers in the system, sampled or not^48^. Estimates of network properties are robust to the inclusion/exclusion of network nodes (Supplementary Fig. 8). Moreover, our data show that the warming experienced by many lakes in this dataset, particularly in the past decade, has shifted network properties and trophic controls to an area of parameter space where slight increases in temperature or phosphorus levels can trigger dramatic changes in the network (Figs. 2 and 4, and Supplementary Fig. 5). Given the stakes for biodiversity and water security, it is currently urgent to forecast future lake ecosystem states, to allow conservation and management by exploring outcomes under different climate scenarios. While the tools used here will enable researchers and stakeholders to measure and predict the complex relationships between network properties, climate change and nutrient supply, our detected trends in decreasing network connectance may have implications for the accuracy of future ecological forecasting^49^.

## Methods

### Data collection

#### Plankton abundance time series

Plankton samples were collected between 1977 and 2020 monthly across ten Swiss lakes (Fig. 1 and Supplementary Table 1). Lake code names are as follows: BAL, Baldegg; BIE, Biel; BRZ, Brienz; GRE, Greifen; HAL, Hallwil; MUR, Murten; NEU, Neuenburg; SEM, Sempach; THU, Thun; and ZHR, Zurich. In lakes Baldegg and Sempach, data from 2010 onwards were excluded from analyses due to irregular plankton sampling after phosphate levels stabilized (that is, bi- or tri-monthly). Samples in all lakes have been collected at identical locations over the years and counted by the same taxonomists for each lake.

Phytoplankton and small zooplankton grazers (that is, rotifers and ciliates) were sampled integrated over the water column in the photosynthetic zone using a Schröder sampler^50^ or at discrete depths, where the lowest depth varied across lakes (Supplementary Table 1). Sampling depth changed in BRZ from 0–20 m to 0–40 m in 2012, in BIE from 0–10 m to 0–15 m in 1999 and to 0–20 m in 2012, in MUR from 0–10 m to 0–15 m in 2012, in NEU from 0–20 m to 0–40 m in 2012, and in THU from 0–20 m to 0–40 m in 2012. Taxa abundances were converted to cells per litre to compare across lakes. In Lake Zurich, the sampling method was changed in 2012 from discrete depth sampling (0, 1, 2.5, 5, 7.5, 10, 12.5, 15, 20, 30, 40, 60, 80, 100, 120, 130, 135 m) to integrated sampling (<20 m, 20–40 m and >40 m of the water column). To compare discrete with integrated samples, we multiplied each discrete sample by a conversion factor (obtained from a year of sampling where both methods were used simultaneously and biomass estimates between samplings were comparable) and aggregated them to match the corresponding integrated samples, for example, multiplied discrete samples within 0–20 m by their corresponding factor and summed them up to match the integrated samples of <20 m. Lake Biel, Baldegg, Murten, Neuenburg, Thun and Zurich sampling did not consider small grazers (ciliates and rotifers).

Large zooplankton was sampled using net-tows going from the bottom of the lake to the surface. Specific details about the lake sampling protocols can be found elsewhere^25,30^. Zooplankton densities were converted to individuals per square metre to compare across lakes. A full taxonomic list of species considered within this study can be found in an open-access data repository linked to this article (10.25678/0007VX). Plankton abundance data were winsorized, where values lying outside the 99% quantile were replaced by the highest values within the 99% quantile using the function Winsorize from the R package DescTools (v.0.99.43). This was done to reduce the power of large outliers without deleting data, because small typos can lead to large outliers in plankton counts. The main trends in connectance and interaction strength were robust to winsorizing (Supplementary Fig. 9).

#### Water temperature and nutrient availability as environmental drivers

Chemical and physical parameters were measured monthly (occasionally bi- or tri-monthly) in the same locations where plankton samples were collected. Samples were taken from the surface to the lake’s bottom at discrete depths. We focused on two main drivers of anthropogenic change in Swiss lakes, water temperature and freely available dissolved phosphate (PO_4_)^51,52^. We used mean water temperature and phosphate concentration over the whole water column. Missing values were estimated using linear interpolation with na_approx from the R package zoo (v.1.8-9). The approximated values ranged between 1 and 268 (Supplementary Table 1). After re-oligotrophication, PO_4_ levels remained constant and often below the detection limit in lakes Biel, Brienz, Hallwil, Murten, Neuchatel and Thun. Sampling for nutrients in those lakes was changed to bi- or tri-monthly early, resulting in 120–268 approximated values.

### Conceptual planktonic network

To understand processes at the network level and control for potential biases in taxa classification across lakes and over time, we aggregated plankton taxa abundances into a conceptual network based on taxonomic classification, body size and feeding behaviour^46^. This allowed us to overcome the limitations of a monitoring frequency lower than the generation time of the organisms, and account for the intrinsic variability of species interactions while reducing the potential effects of taxonomic misclassification^53^. The dynamics of trophic guilds occur at the scale of months, as opposed to the dynamics of taxa, which occur at the scale of days, and thus well represent seasonal and interannual network transitions^32,46^.

Our conceptual network consisted of up to 15 nodes (guilds) across three trophic levels of the food web, containing large invertebrate predators, omnivores, large herbivore grazers, small grazers, mixotrophs and primary producers. In lakes Biel, Brienz, Murten, Neuenburg, Thun and Zurich, we only had 13 guilds because of missing counts for rotifers and ciliates. We conducted a sensitivity analysis where we excluded rotifers (Ro) and ciliates (Ci) from Lake Baldegg, Greifen, Hallwil and Sempach data. Connectance and interaction strength were similar with and without rotifers and ciliates (Supplementary Fig. 8). Because we could not differentiate between calanoid and cyclopoid nauplii nor their juvenile stage, and thus had insufficient information on their feeding behaviour, nauplii were excluded from our study. Small single-cell cyanobacteria were excluded as well, as most taxa are below the size-detection limits of traditional microscopy.

The relationships (links) between nodes can be trophic (classic predator–prey relationship), non-trophic (that is, mutualisms and competition) or hybrid, where guilds can have trophic or non-trophic relationships (that is, mixotrophic flagellates; Fig. 1c). All links are bi-directional (in both directions), which means trophic and hybrid links can go up the network (bottom-up), that is, from a primary producer to a grazer, as well as down the network (top-down), that is, from a grazer to a primary producer (Fig. 1c).

### Data analysis

Chaos and nonlinear dynamics are ubiquitous in plankton communities, making linear statistical approaches unfit to study long-term changes in their network properties^54^. In particular, nonlinear dynamics can obscure correlations between variables, making causal links undetectable with classical statistical methods. Equation-free approaches, such as EDM, which can recover dynamics from empirical data, overcome this limitation and offer a promising non-parametric way to study nonlinear systems (see http://tinyurl.com/EDM-intro for a brief video introduction). EDM, which is rooted in state-space reconstruction, can be used to determine the number of dimensions required to describe a system (best embedding)^55,56^, quantify the nonlinearity of time series^57–59^, forecast future system states^55,60–62^, infer causality between two variables^34^ and quantify how relationships (interactions) between variables change with changing system state^19^. A more in-depth description of EDM can be found in the Supplementary Methods (extended). We expanded the classical EDM framework by adding a temporal component to CCM (studying local correlations among observations and predictions within a moving window)^34^ and using the predictive skill rho (corrected for seasonality) as a proxy for how strongly a network node is affected and/or affects another node. Moreover, we used S-maps to explore the interactive effects of water temperature and nutrient levels on network properties^57^.

### Reconstructing a time-varying causal network using CCM

CCM is a ‘nonlinear causality test’ that estimates the extent to which changes in one variable affect changes in another by measuring cross-prediction (as explained herewith). Consider two variables, V1 (for example, phytoplankton) and V2 (for example, large herbivores or temperature). We want to know whether and how strongly V1 is impacted by V2; that is, V1 ← V2. This is determined by measuring how much V2 has impacted the dynamics (time series) of V1—how much information about V2 has been imprinted in the time series of V1. This information allows one to use V1 to estimate the states of the driver V2, a process known as cross-mapping between variables^34^. The stronger the signature (causal impact in the affected variable), the better the cross-map estimate. To do this in the R package rEDM, we would call V1 xmap V2, where again the direction of effect we are testing is V1 ← V2. Note that the time series require added placeholders for missing values to ensure having evenly spaced monthly data. As the time series used are on different scales (for example, temperature measurements and abundance data), we rescale them using the function scale in the R package base (v.4.1.0).

#### Embedding dimension

We use simplex from the R package rEDM (v.0.7.5) to define the best embedding dimension for V1 using simplex projection (Supplementary Information). The embedding dimension was run over *E* = 2:15. Time lag and prediction horizon were set to 1 month. The number of nearest neighbours used to make predictions are set to *E* + 1. Forecasting was done using leave-one-out cross-validation and the best embedding was selected based on maximizing the forecasting skill rho (Supplementary Table 3).

#### Convergence test

We tested the convergence of V1 xmap V2 by comparing the predictive power of using 20% and 50% of the data, respectively. This was done with 100 consecutive random subsets of the time series. The ideal embedding dimension was defined for V1 based on forecasting with simplex projection (see above and Supplementary Table 3), while the time lag tp was kept at 0. CCM was run with the function ccm from the R package rEDM (v.0.7.5). Convergence was considered true if rho_50%_ > rho_20%_ for the 100 subsets, determined by a one-sided *t*-test (95% quantile).

#### Local cross-mapping-skill (rho)

If the convergence test was significant, we performed CCM between V1 and V2 this time using the maximum library (whole time series) and tp = −1. Using the predictions from the CCM output, we calculated local rhos, that is, the correlation between observation of V2 and predictions of V2 (using V1’s attractor) within moving windows (*n* = 60 months, sliding 1 month forward at a time). This resulted in a time series of rhos (forecast skills).

#### Seasonal surrogates

The local rhos (rho_originalTS_) were then compared with rhos from 100 random seasonal surrogate time series (rho_surrogateTS_) for each time window (time point *t*_*x*_). We considered the link V1 ← V2 at time point *t*_*x*_ as significant if 95% of the times rho_originalTS_ > rho_surrogateTS_. If the link was significant, we estimate the strength of V1 ← V2 at *t*_*x*_ by removing the seasonal component from the local rho_originalTS_, that is, rho_originalTS_ − mean(rho_surrogateTS_), the average local rho of the 100 surrogate time series. Negative rhos were always set to 0.

#### Network links

To calculate network connectance, we summed all causal links (passed the surrogate test) per lake and date (month) and divided them by the total possible links for this network (based on the conceptual network in Fig. 1c and convergence test). We obtained connectance (%), the number of connected nodes, for this time point and a time series of connectance per lake (Fig. 2a).

#### Taxa interaction strength

We calculate the mean strength of links across nodes per date and lake. This resulted in average link strength for this time point and a time series of average link strength per lake (Fig. 2b). Taxa interaction strength over time and across lakes (Extended Data Fig. 6) was calculated by estimating the average strength of each link and multiplying it by its prevalence over time (per lake), that is, corrected the strength for how often it occurred in the time series, and then averaged across lakes.

#### Environment effect on guilds

To get at the strength of water temperature and phosphate effects over time on each guild’s abundance (Extended Data Fig. 6), we calculated the local cross-mapping-skill rho and compared it with a value obtained by a seasonal null model. We averaged the strength of water temperature and phosphate effects for each node and multiplied by its prevalence over time, and then averaged across lakes (analogous to calculating interaction strengths between guilds over time across lakes).

#### Feedback between temperature and nutrients

To test for a causal relationship (feedback) between water temperature and phosphate concentration (Extended Data Fig. 2), we used CCM on the whole time series and performed a convergence test (*n* = 100) and seasonal surrogate test (*n* = 100). If both the convergence test (rho_50%_ > rho_20%_) and seasonal surrogate test (>95% of times rho_originalTS_ > rho_surrogateTS_) passed, we considered an effect as significant (lake displayed as points in Extended Data Fig. 2). To get a robust estimation of the effect’s magnitude (that is, filter out single episodic events and diminish the power of outliers), we multiplied the strength of the effect at each time point by its prevalence over time (per lake), that is, we corrected the strength of the causal effect by how often a significant effect occurred in the time series. The resulting value was plotted on the *y* axis in Extended Data Fig. 2.

#### Trophic controls

We summed up all causal links going up (bottom-up) and down (top-down) the food web (that is, trophic and hybrid links) per time point and lake, and divided them by all the total possible bottom-up or top-down links for this network (Fig. 1c). Moreover, we averaged the strength of all significant bottom-up and top-down links per time point and lake. Then we calculated the difference between realized top-down and bottom-up links (that is, top-down connectance − bottom-up connectance) and top-down and bottom-up strength (that is, top-down link strength − bottom-up link strength). This resulted in a time series of changes in trophic controls over time, whereas a value >0 indicated top-down and <0 bottom-up control (Fig. 4a,b). If there were no significant bottom-up and/or top-down links at a given time point, connectance was set to 0 and strength to NA (unknown).

#### Interaction types

We summed up all trophic, non-trophic and hybrid links (according to Fig. 1c) per time point and lake, and divided them by all total possible links per interaction type. Then we averaged connectance and interaction strength for trophic, non-trophic and hybrid links per time point across lakes. This resulted in a time series of connectance (%) and strength of trophic, non-trophic and hybrid links (Supplementary Fig. 7). We compared connectance (%) and strength of interaction types using a Wilcoxon test, a non-parametric method for testing if samples originate from the same distribution (Fig. 5).

### Scenario exploration using multivariate S-maps

We used multivariate S-maps to model network properties and extract their relationship with phosphate levels and water temperature. S-maps compute a unique locally weighted linear regression to make a forecast at each point in time when closer points on the attractor are given a higher weight. The strength of weighting is controlled by the parameter theta and indicates the degree of nonlinearity and state dependency. Each regression provides a set of coefficients that define relationships (dynamics) between variables at each unique state. These coefficients were used to estimate (predict) each network property at varying levels of temperature and phosphate (Figs. 2 and 4c,d, and Supplementary Figs. 4 and 5). To account for important differences in the morphometry of lakes, which influence these ecosystems’ responses to changes in nutrient inputs and warming, we included depth at the sampling site and lake total water volume in the S-map models^25^. Confidence in the predictions can be influenced by the parameter space covered by the lake time series, for example, less confidence in the predictions for combinations of high water temperature and phosphate levels (Supplementary Fig. 10).

We ran the S-map models using rEDM (v.0.7.5) and the function block_lnlp for 100 random subsets, using 50% of the data and averaged (mean) the predictions. The variance was calculated by estimating the standard deviation among the 100 predictions. Environmental drivers were smoothed within 60-month moving windows to match the temporal scale of modelled network properties. We chose 100 values for temperature and phosphate levels (each), ranging from the minimum to the maximum values observed across all lakes. This resulted in a grid of 10,000 model predictions (Figs. 2 and 4c,d, and Supplementary Figs. 4 and 5**)**. Methods within the function were set to ‘s-map’ and the exclusion radius to 12 to avoid the high temporal autocorrelation caused by the moving windows. Theta was selected to maximize predictive skill rho when varied over a list of values (0, 0.0001, 0.0003, 0.001, 0.003, 0.01, 0.03, 0.1, 0.3, 0.5, 0.75, 1.0, 1.5, 2, 3, 4, 6 and 8) and tp set to 0.

### Reporting summary

Further information on research design is available in the Nature Portfolio Reporting Summary linked to this article.

## Online content

Any methods, additional references, Nature Portfolio reporting summaries, source data, extended data, supplementary information, acknowledgements, peer review information; details of author contributions and competing interests; and statements of data and code availability are available at 10.1038/s41558-023-01615-6.

## Supplementary information

Supplementary Information Supplementary Figs. 1–10, Tables 1–3 and Methods (extended).

Reporting Summary

## Data Availability

An overview of the taxonomic list and guild classification and the guild abundances for each lake (Supplementary Fig. 1), including environmental drivers, can be found in an open-access data repository linked to this article (10.25678/0007VX)^63^.

## Code availability

The R code to reproduce the analysis and figures is available in an open-access data repository linked to this article (10.25678/0007VX)^63^. Current software versions of EDM tools are available in Python at https://pepy.tech/project/pyEDM and in R on CRAN.
