# Supplementary material for: Disruption of ecological networks in lakes by climate change and nutrient fluctuations
Source: Nat Clim Chang. 2023 Mar 23;13(4):389–96. doi: 10.1038/s41558-023-01615-6 (PMC10079529; doi:10.1038/s41558-023-01615-6)
Supplement: Supplementary file 1 — Supplementary Figs. 1–10, Tables 1–3 and Methods (extended). [file 41558_2023_1615_MOESM1_ESM.pdf]

nature climate change

Article

https://doi.org/10.1038/s41558-023-01615-6

Disruption of ecological networks in lakes by climate change and nutrient fluctuations

In the format provided by the authors and unedited

Table of Contents

Supplementary figures

1.	Fig. S1 Time series of plankton guilds	2-11
2.	Fig. S2 Node richness is constant in all lakes	12
3.	Fig. S3 Sensitivity analysis of cross-mapping window size	13
4.	Fig. S4: Lake-specific responses of network properties	14
5.	Fig. S5 S-map results for predicting network properties	15-23
6.	Fig. S6 Spearman-rank correlation between top-down and bottom-up control	24
7.	Fig. S7 Temporal changes in interaction type	25-26
8.	Fig. S8 Sensitivity analysis inclusion/exclusion of small grazers	27
9.	Fig. S9 Sensitivity analysis winsorizing taxa abundance data	28
10.	Fig. S10 Availability of temperature and phosphate concentration data	29

Supplementary tables

1.	Tab. S1 Lake metadata	30
2.	Tab. S2 S-map prediction accuracy	31-32
3.	Tab. S3 Best embedding dimension	33

Methods (extended) **34-43**

References **43-45**

Lake Baldegg (BAL)

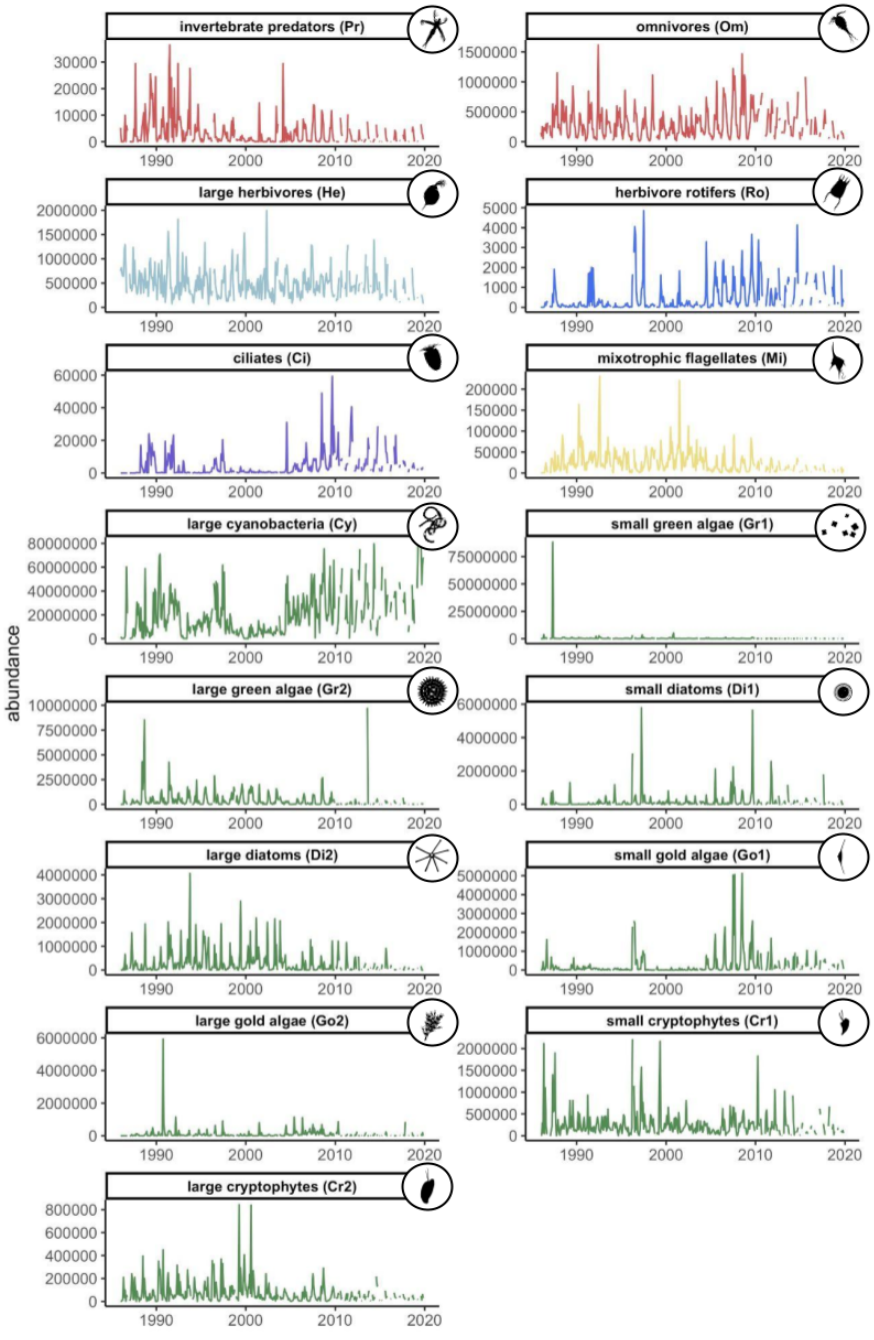

Lake Biel (BIE)

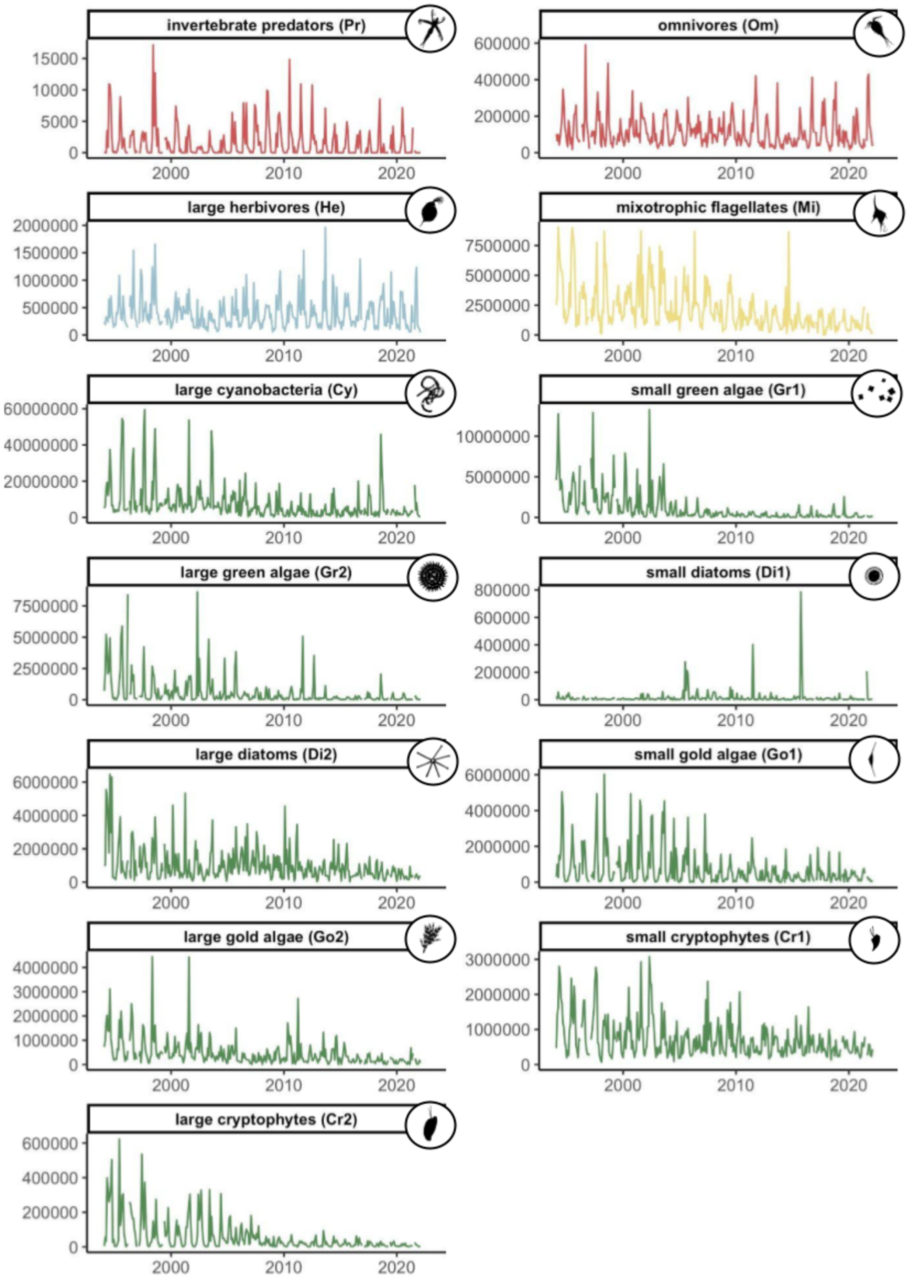

Lake Brienz (BRZ)

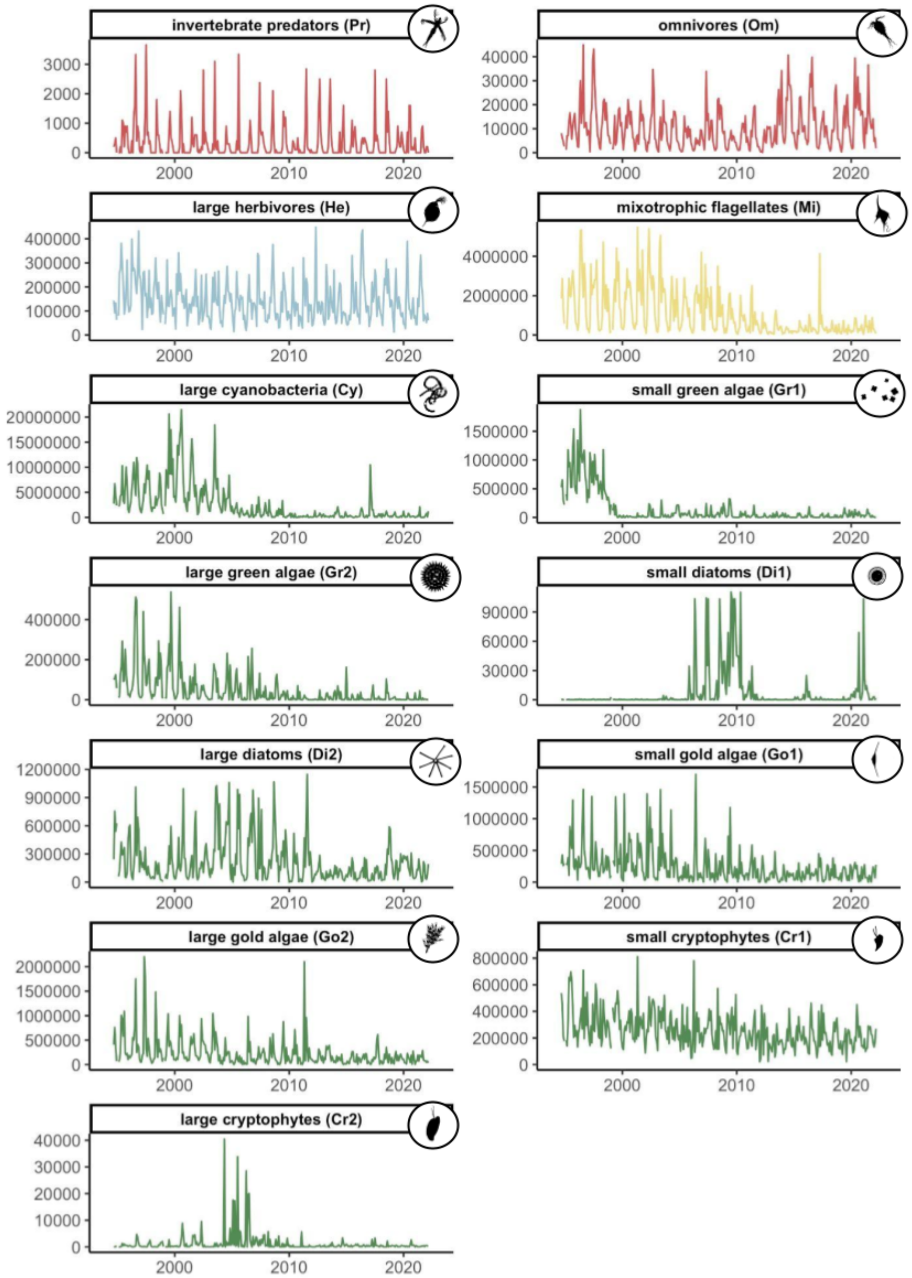

Lake Greifen (GRE)

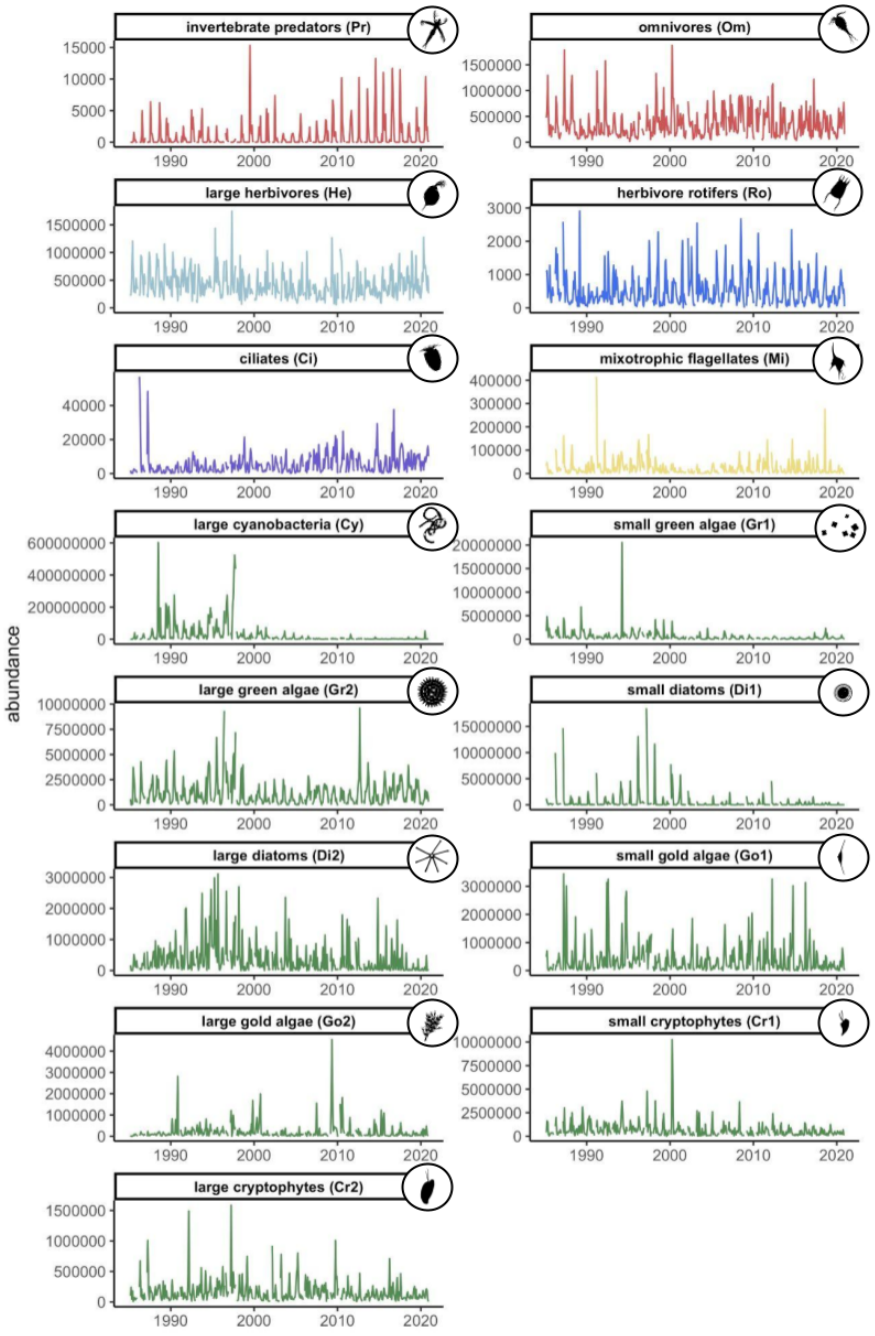

Lake Hallwil (HAL)

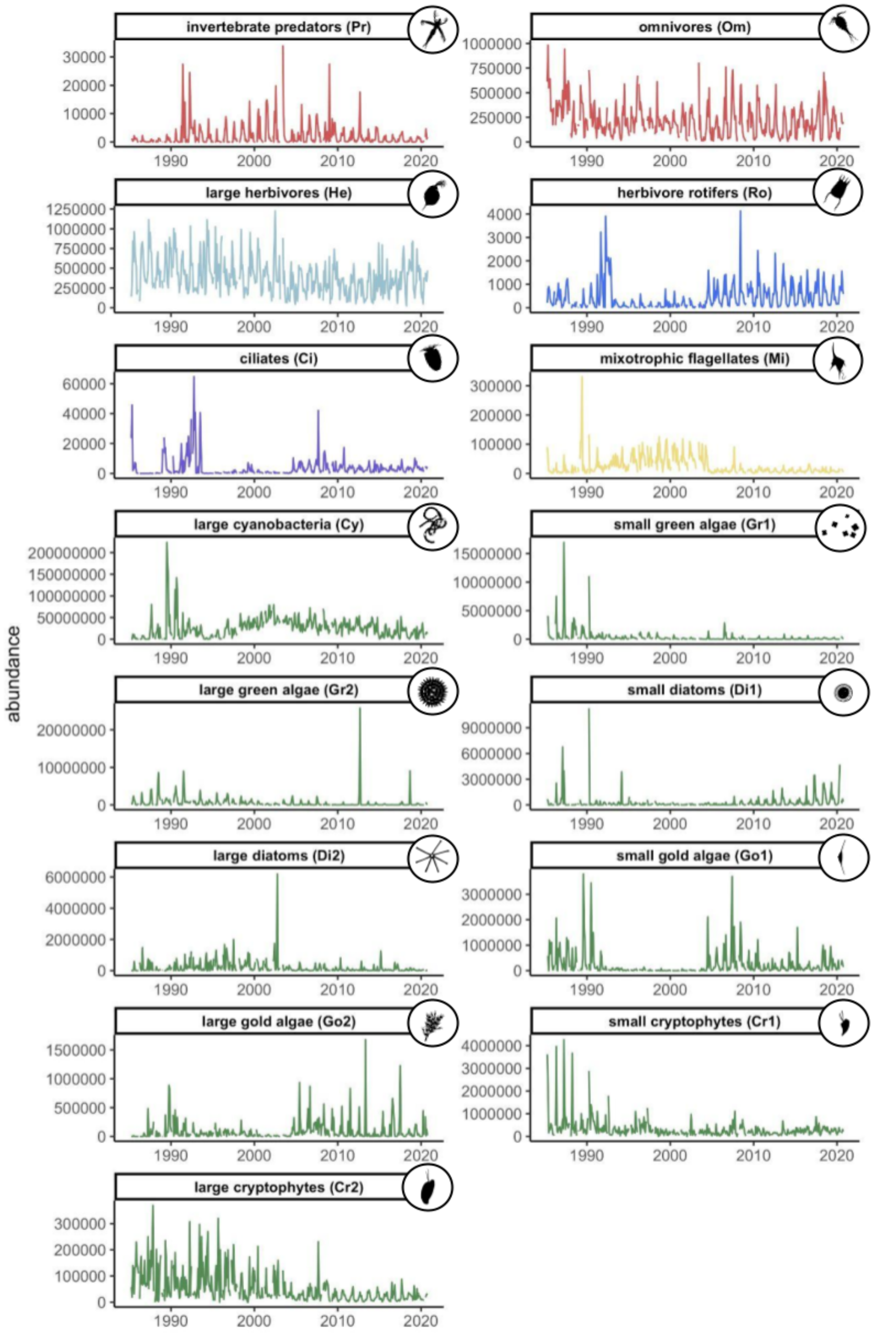

Lake Murten (MUR)

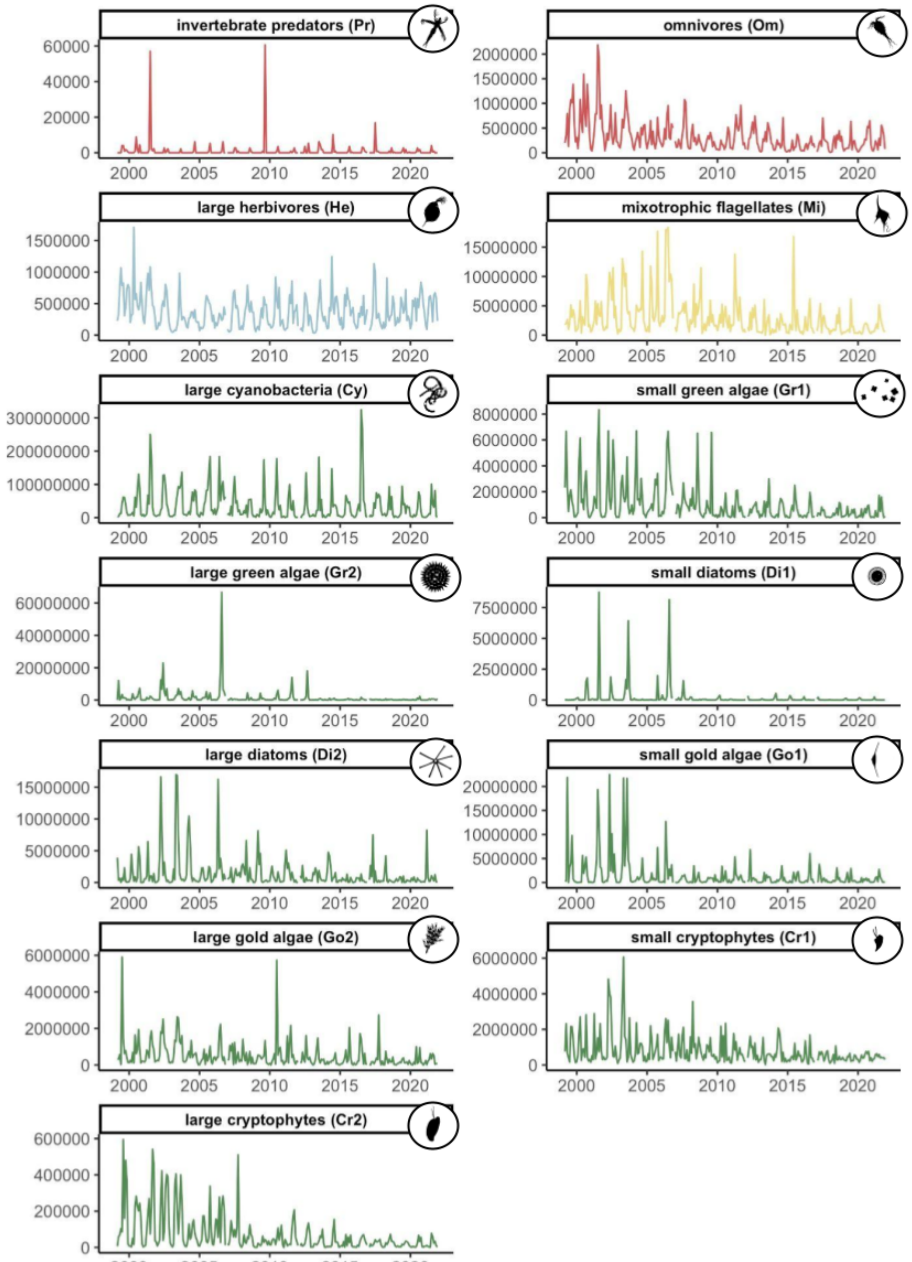

Lake Neuenburg (NEU)

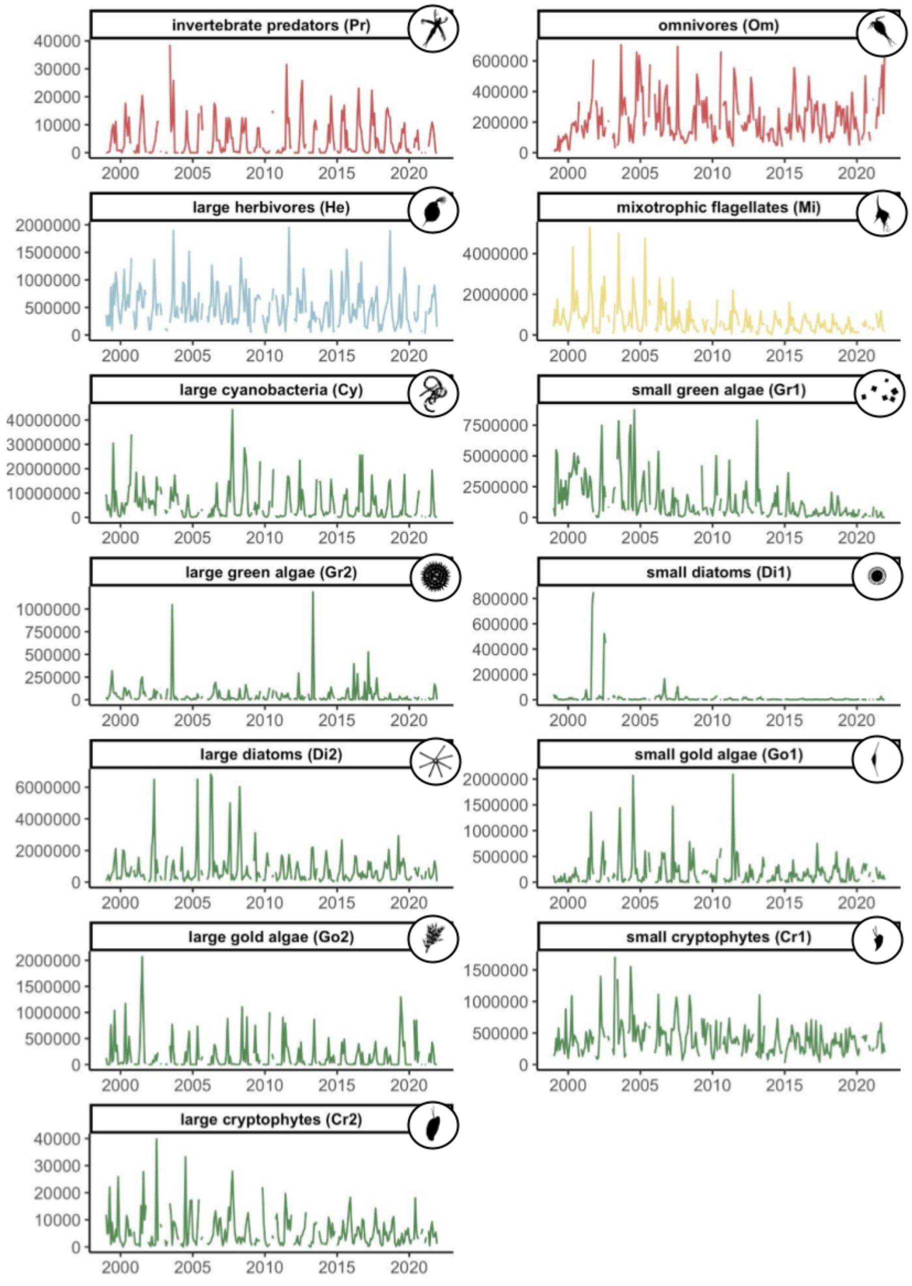

Lake Sempach (SEM)

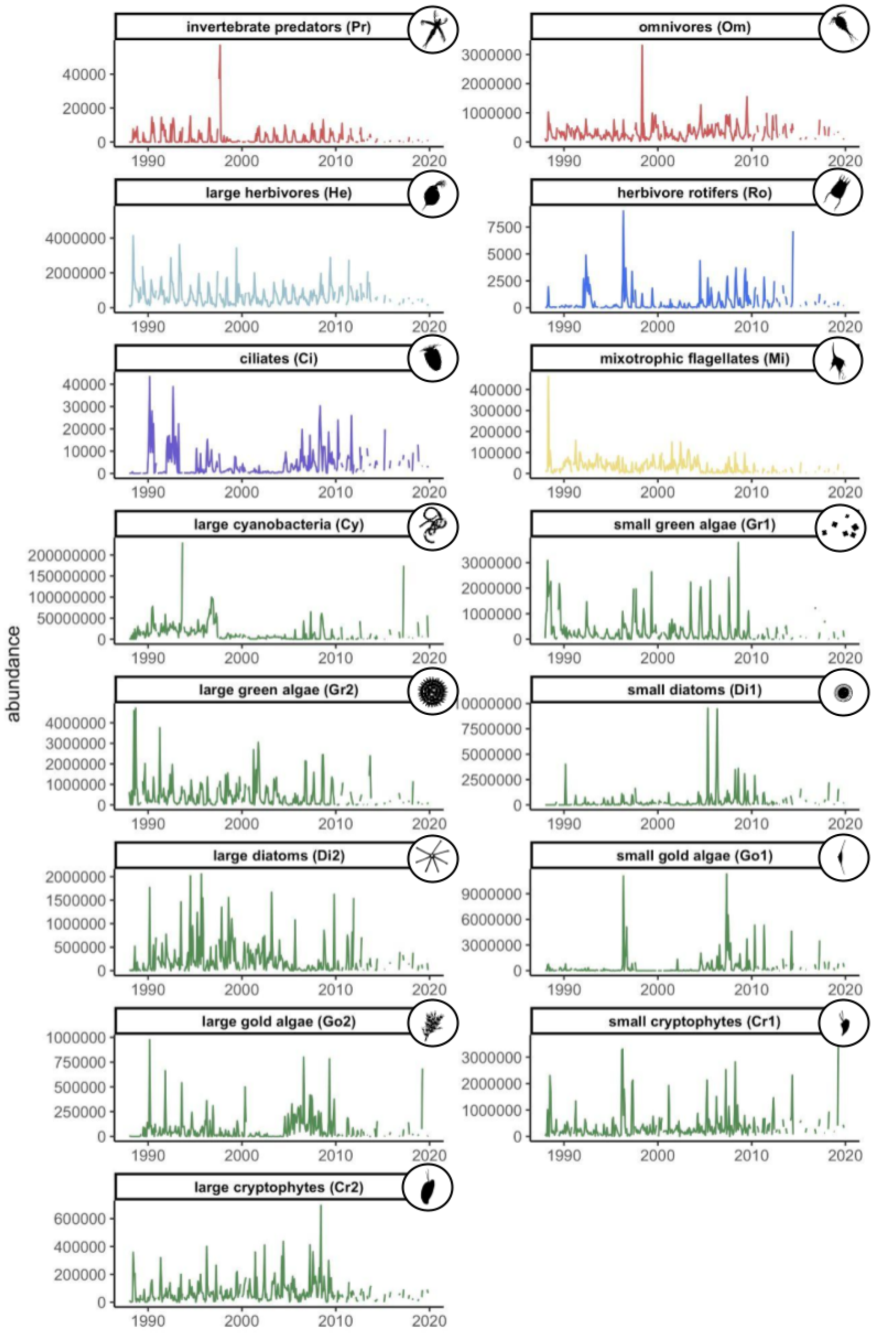

Lake Thun (THU)

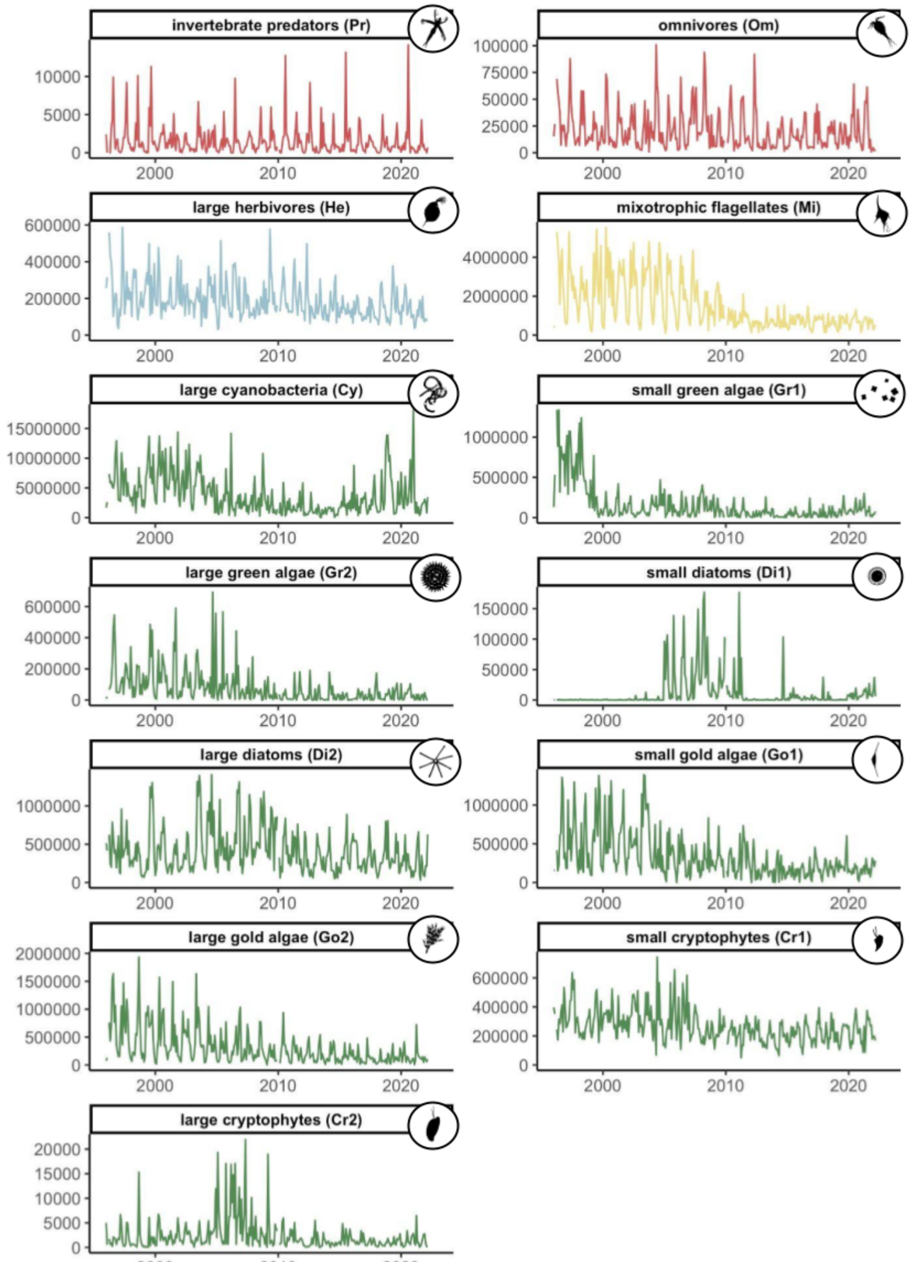

Lake Zurich (ZHR)

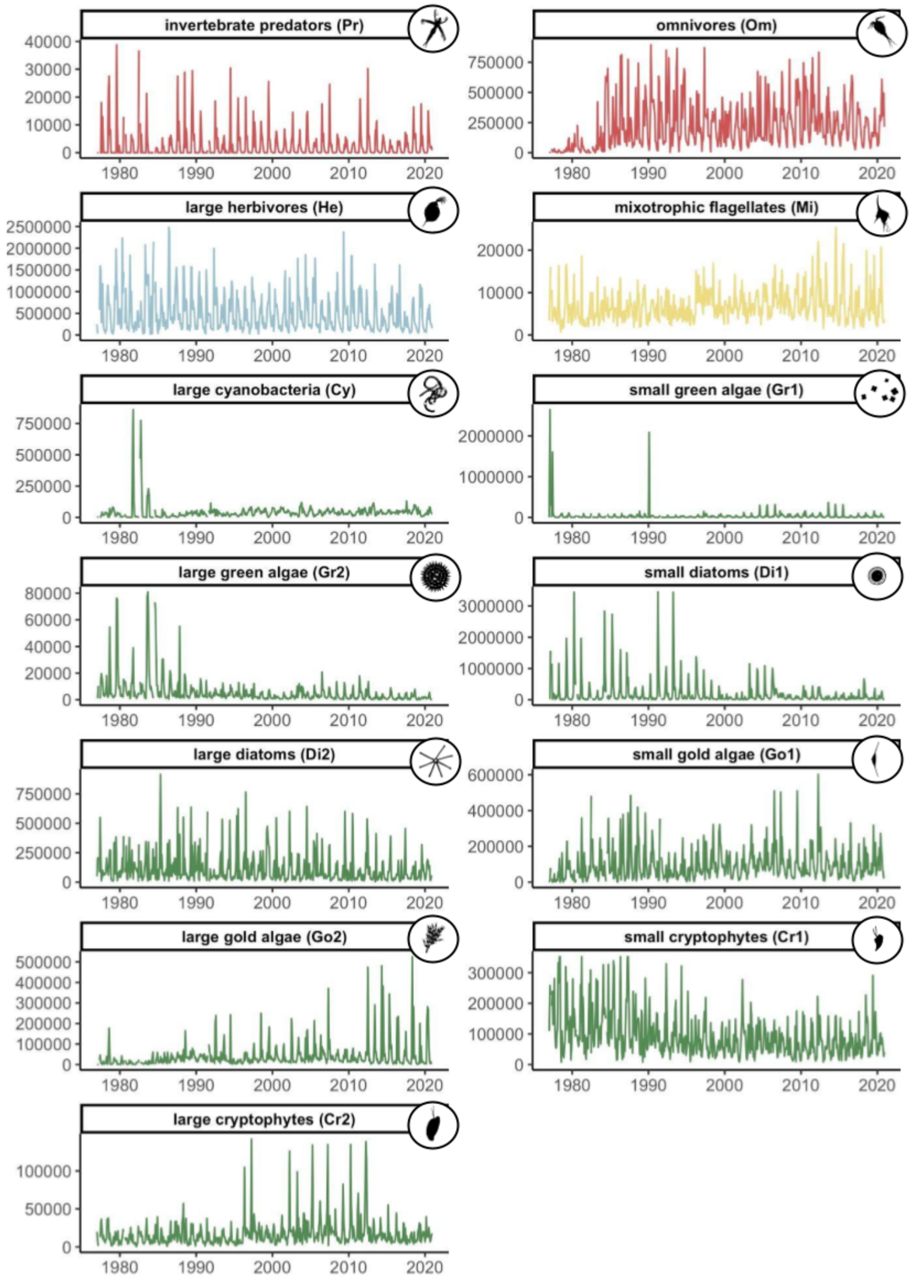

Fig. S1 Time series of plankton guilds (nodes in the network). Abundances of He-Pr are in indv./m2 while Ro-Mi and Cy-Cr2 are in indv./L. He-Pr has been collected over the whole water column, whereas Ro-Mi and Cy-Cr2 were sampled in the photosynthetic zone (**Tab. S1**). Lake BAL, BIE, BRZ, MUR, NEU, THU and ZHR did not consider small grazers (Ro, Ci) during their sampling.

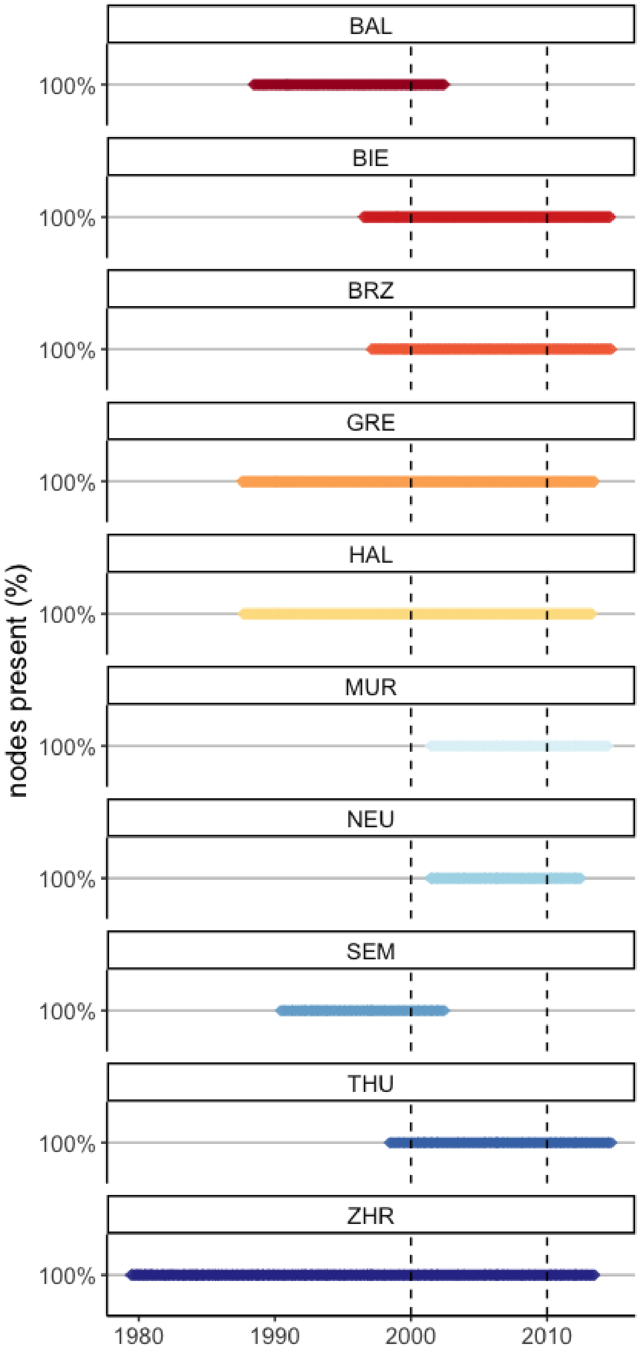

Fig. S2 Node richness is constant in all lakes. Node richness is calculated as the percentage of nodes with an abundance >0 during the 60-month moving window used for the ccm analysis. Points show single measurements in time.

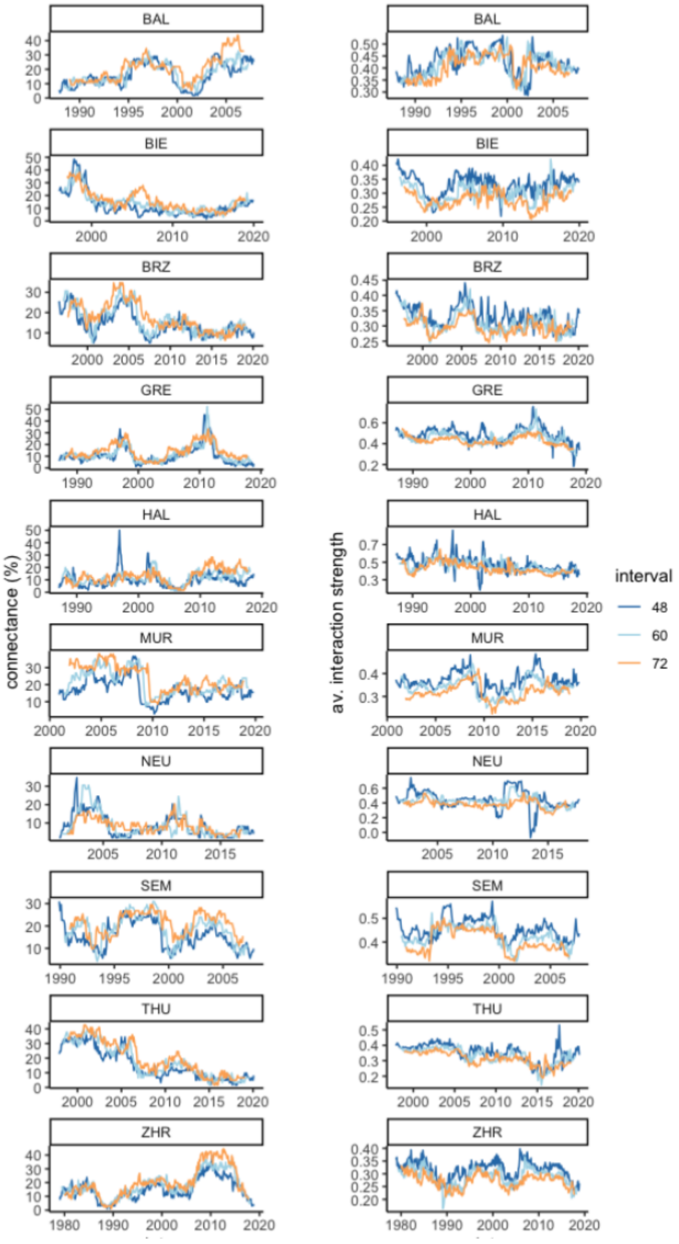

Fig. S3 Sensitivity analysis of cross-mapping window size (48, 60, and 72 months). [a] connectance (%) and **[b]** link strength (estimated from the predictive skill rho, Pearson's correlation between observations and predictions, corrected for seasonality). The main trends are robust to the choice of size of the cross-mapping window.

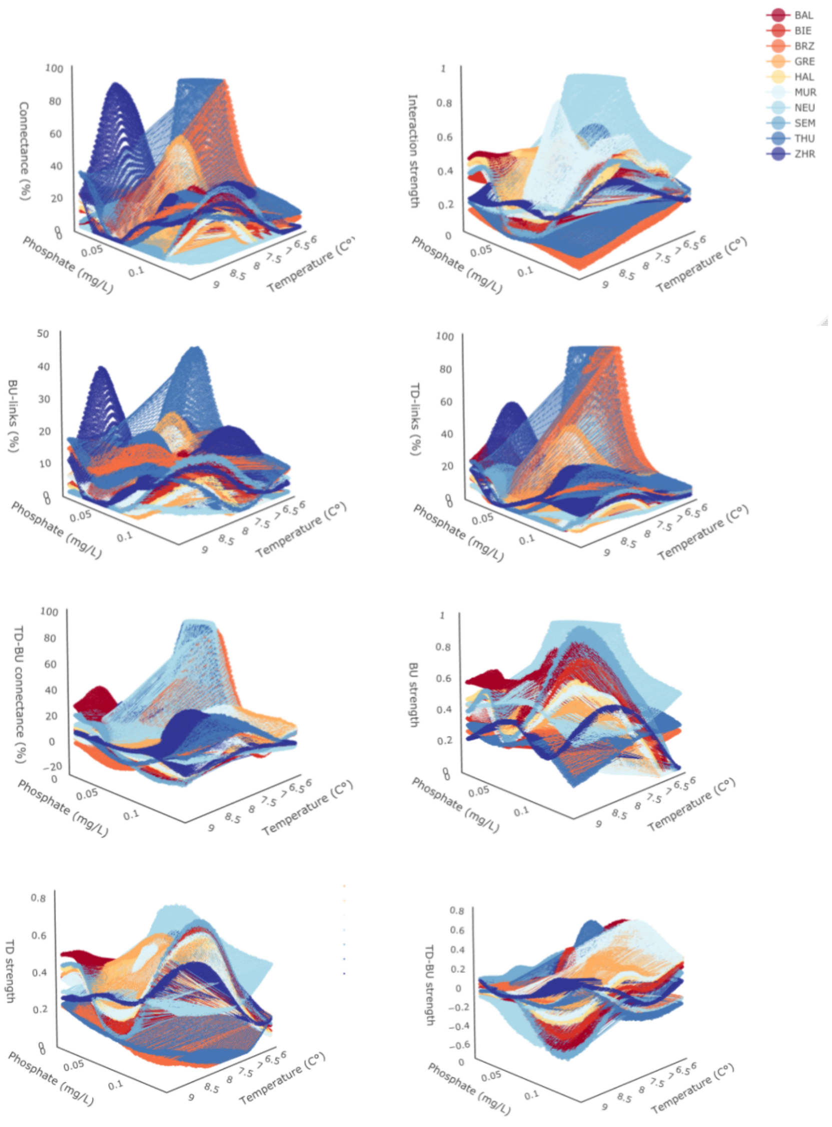

Fig. S4: Lake-specific responses of network properties to changes in water temperature and phosphate levels for the ten lakes. Network properties were modeled using s-maps based on water temperature, dissolved phosphate and a lake's depth and volume. We then predicted them over a gradient of phosphate (x-axis1) and temperature (x-axis2). Network properties are plotted on the z-axis. Colors represent the different lakes.

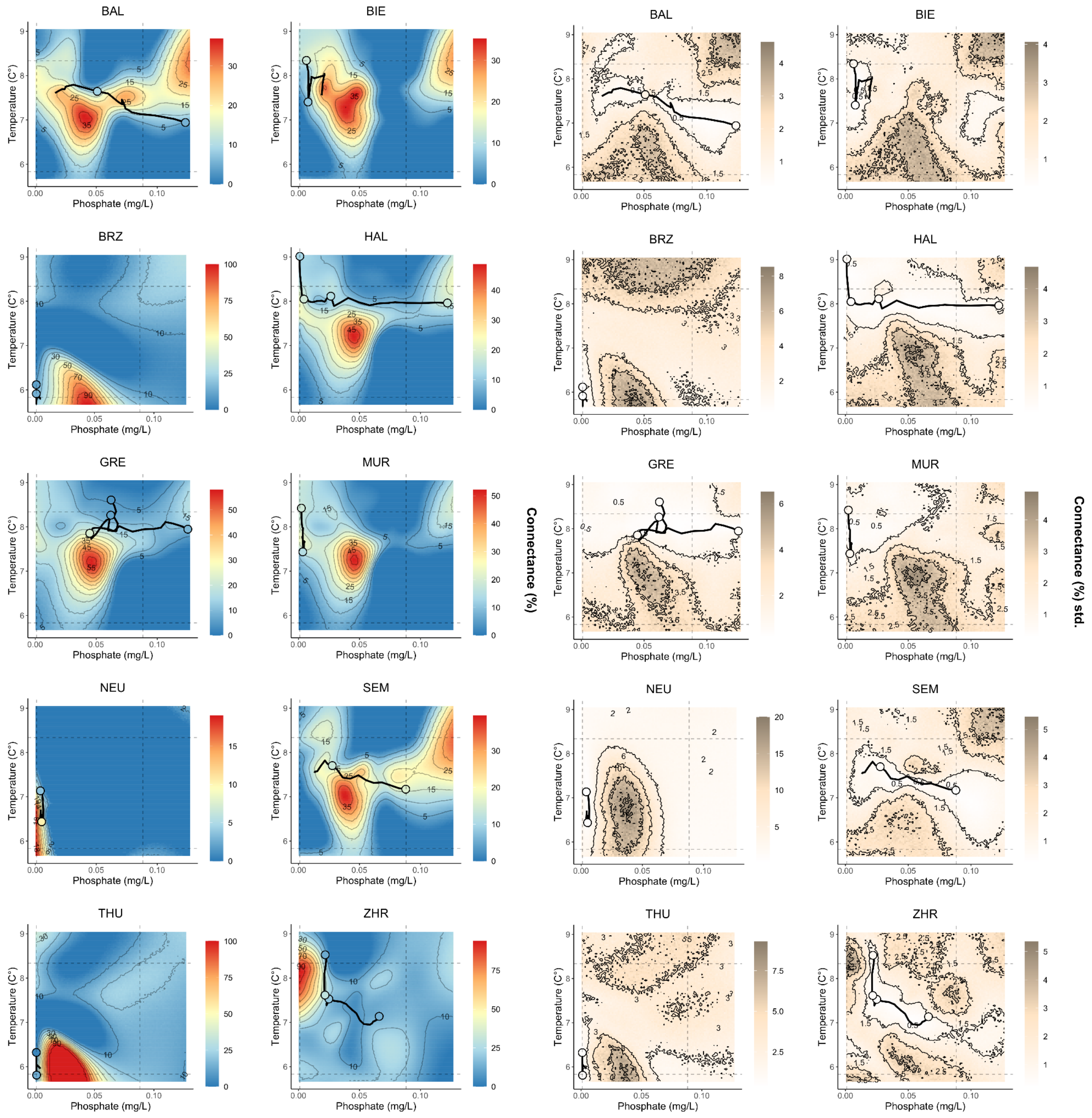
BAL
BIE
BRZ
HAL
GRE
MUR
NEU
SEM
THU
ZHR
Temperature (C°)
Phosphate (mg/L)
Connectance (%)
Connectance (%) std.

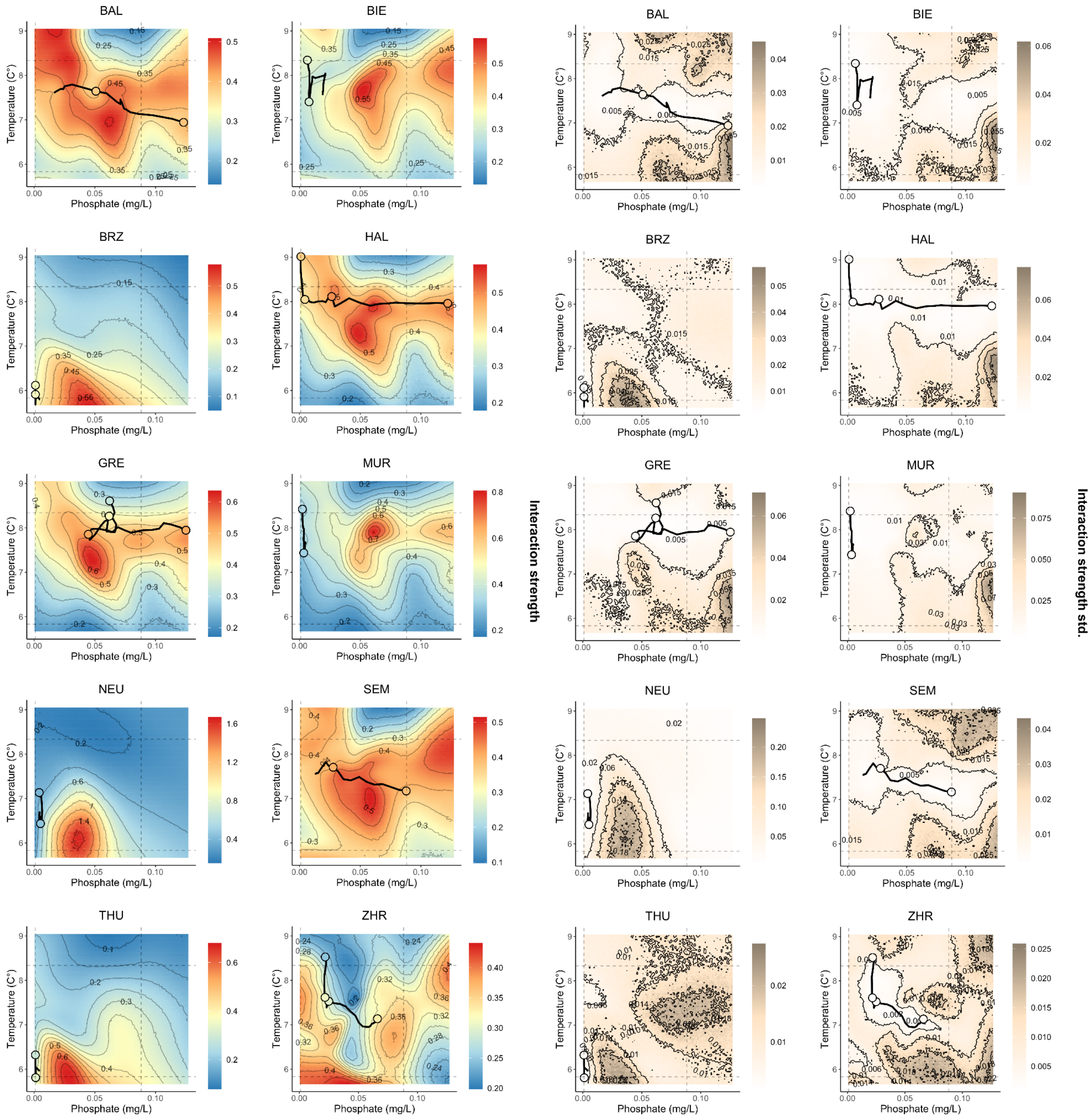
BAL
BIE
BRZ
HAL
GRE
MUR
NEU
SEM
THU
ZHR
Temperature (C°)
Phosphate (mg/L)
Interaction strength
Interaction strength std.

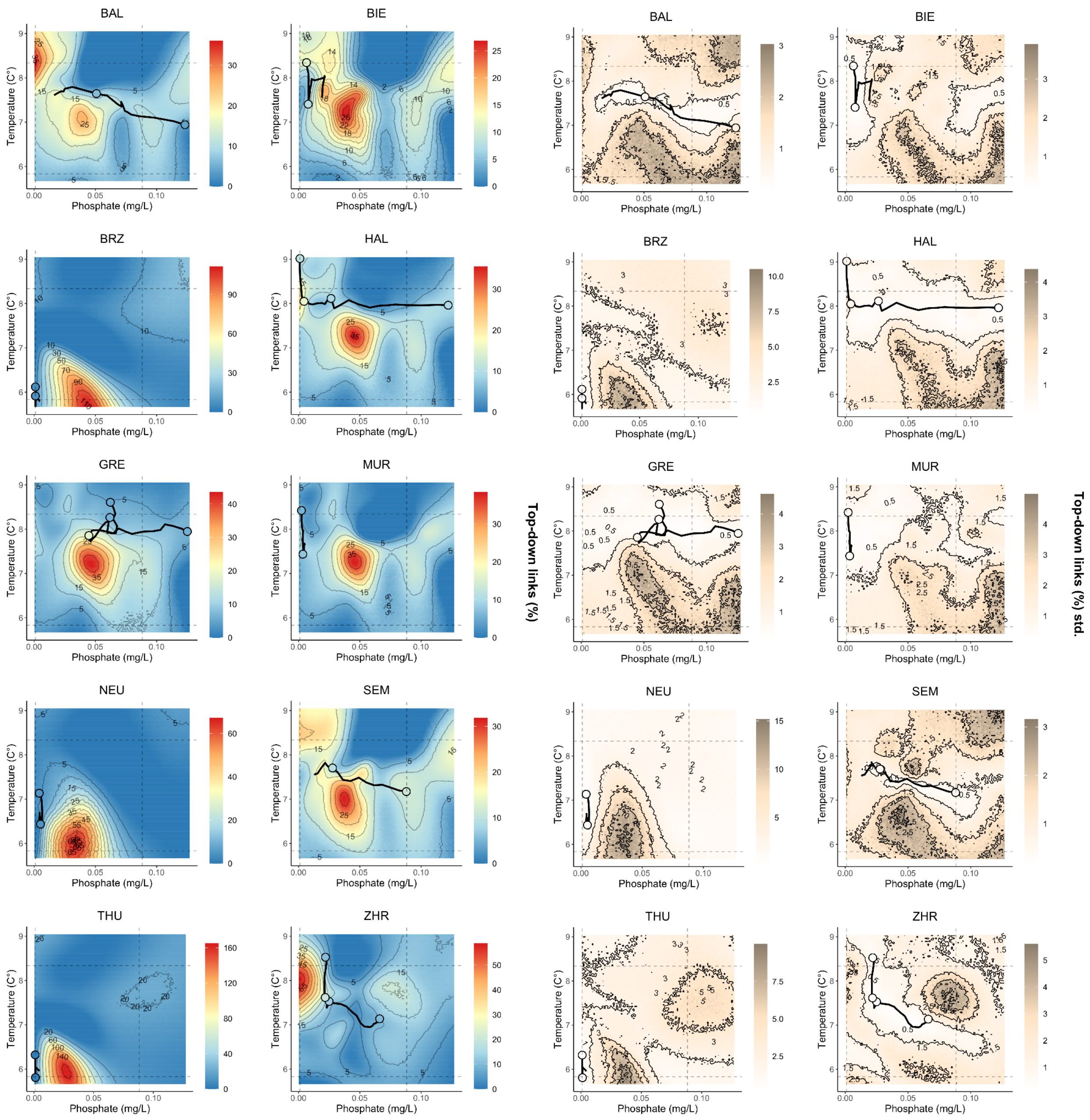
BAL
BIE
BRZ
HAL
GRE
MUR
NEU
SEM
THU
ZHR
Temperature (C°)
Phosphate (mg/L)
Top-down links (%)
Top-down links (%) std.

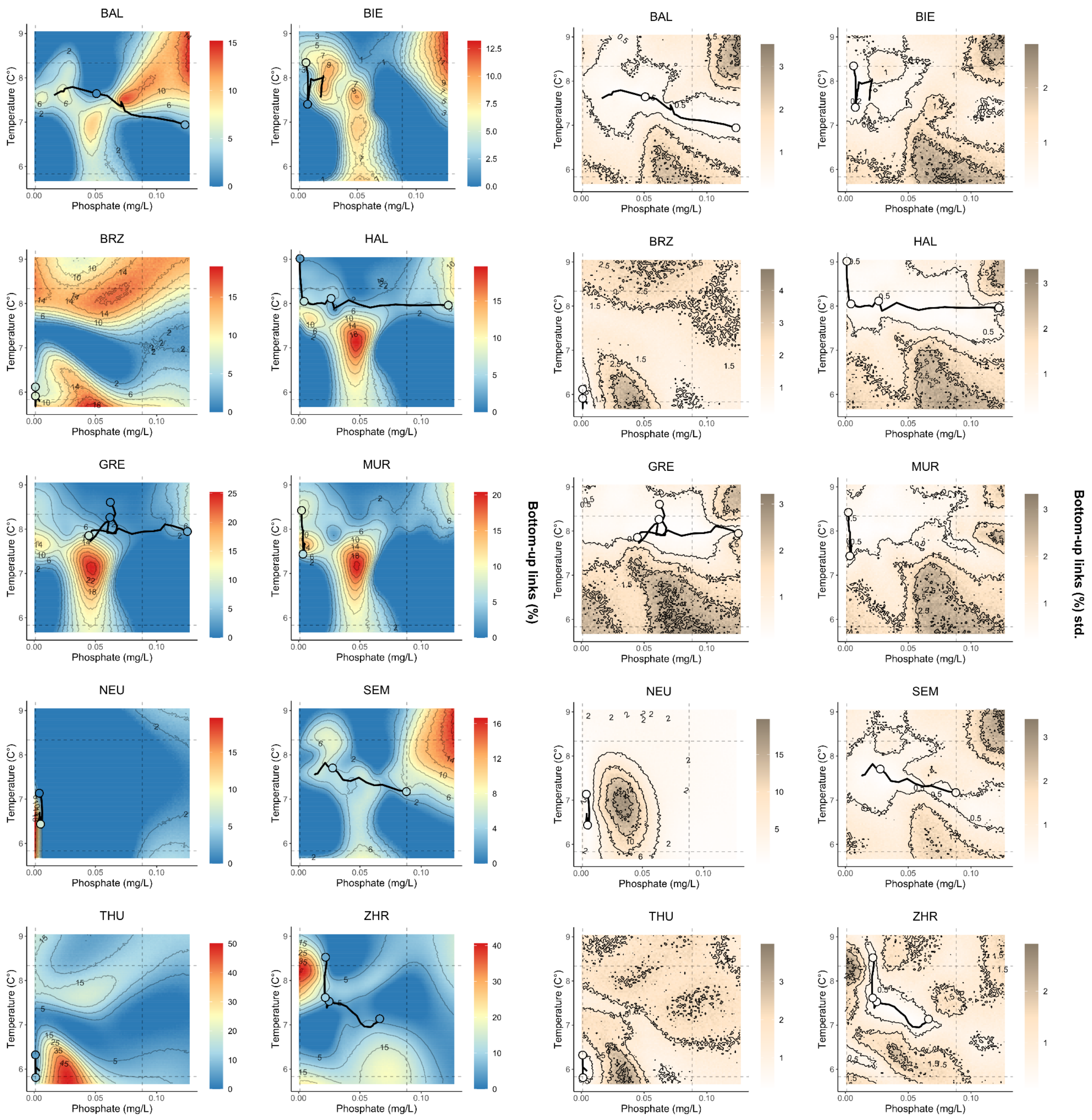
BAL
BIE
BRZ
HAL
GRE
MUR
NEU
SEM
THU
ZHR
Temperature (C°)
Phosphate (mg/L)
Bottom-up links (%)
Bottom-up links (%) std.

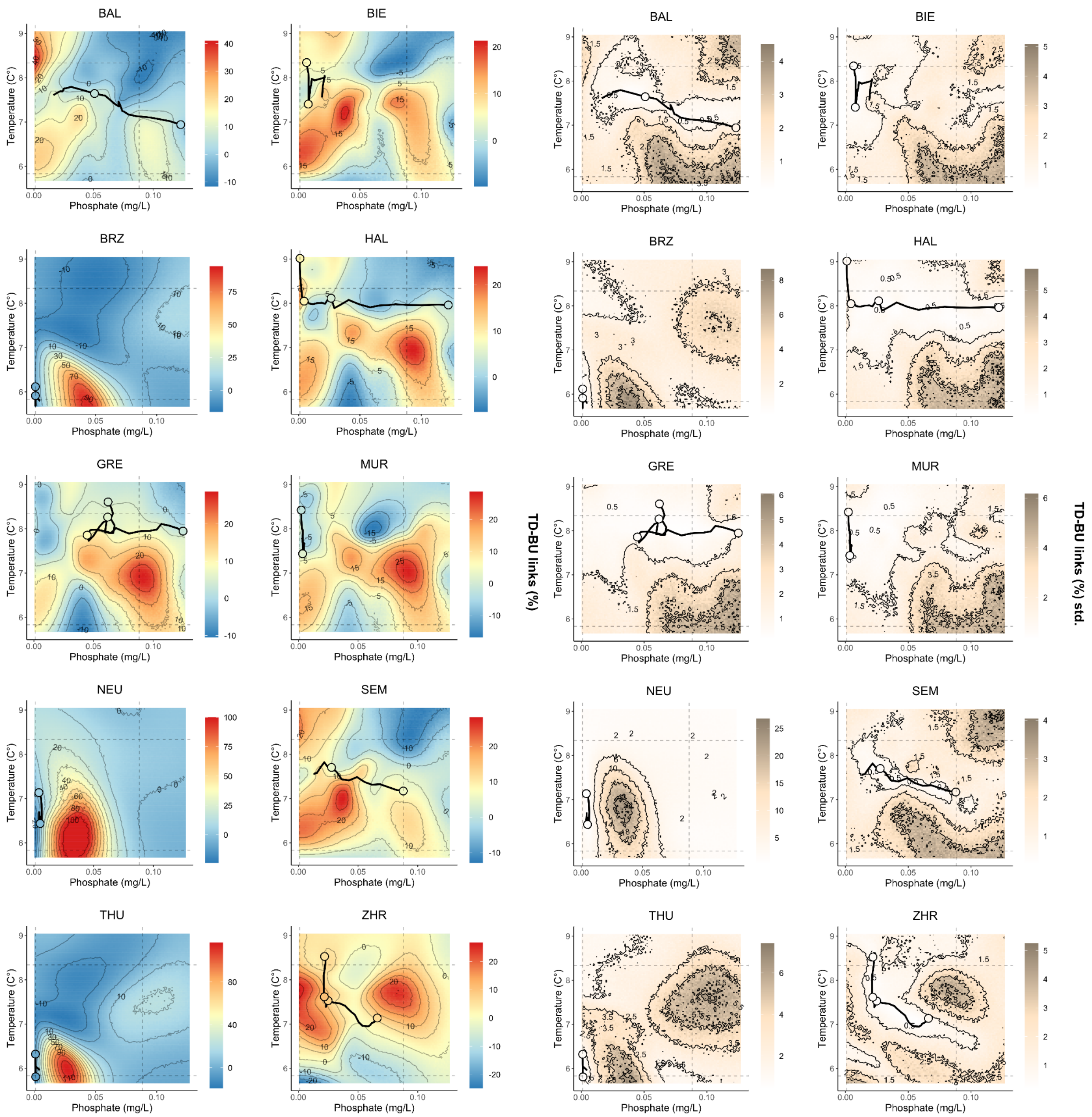
BAL
BIE
BRZ
HAL
GRE
MUR
NEU
SEM
THU
ZHR
Temperature (C°)
Phosphate (mg/L)
TD-BU links (%)
TD-BU links (%) std.

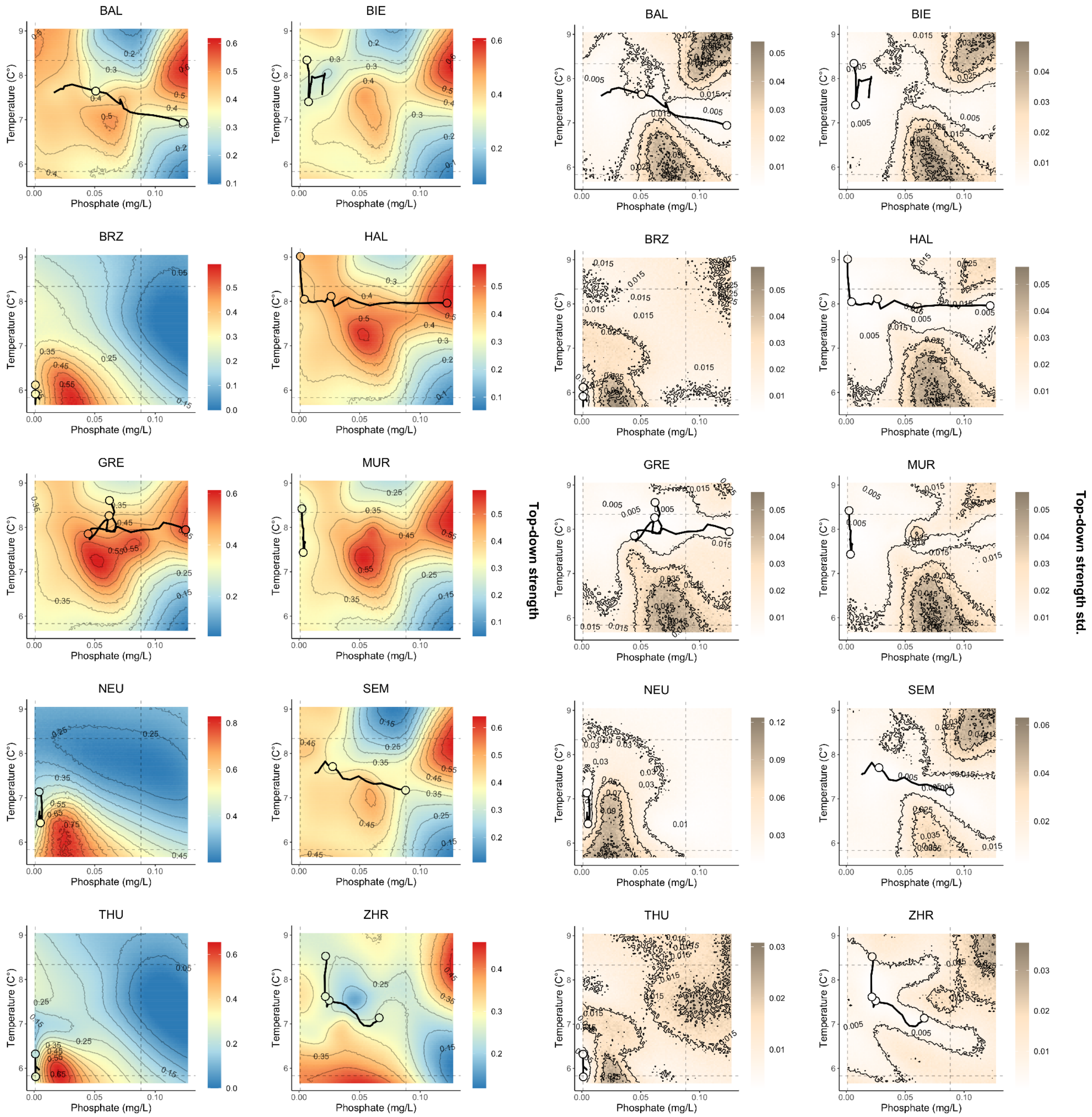
BAL
BIE
BRZ
HAL
GRE
MUR
NEU
SEM
THU
ZHR
Temperature (C°)
Phosphate (mg/L)
Top-down strength
Top-down strength std.

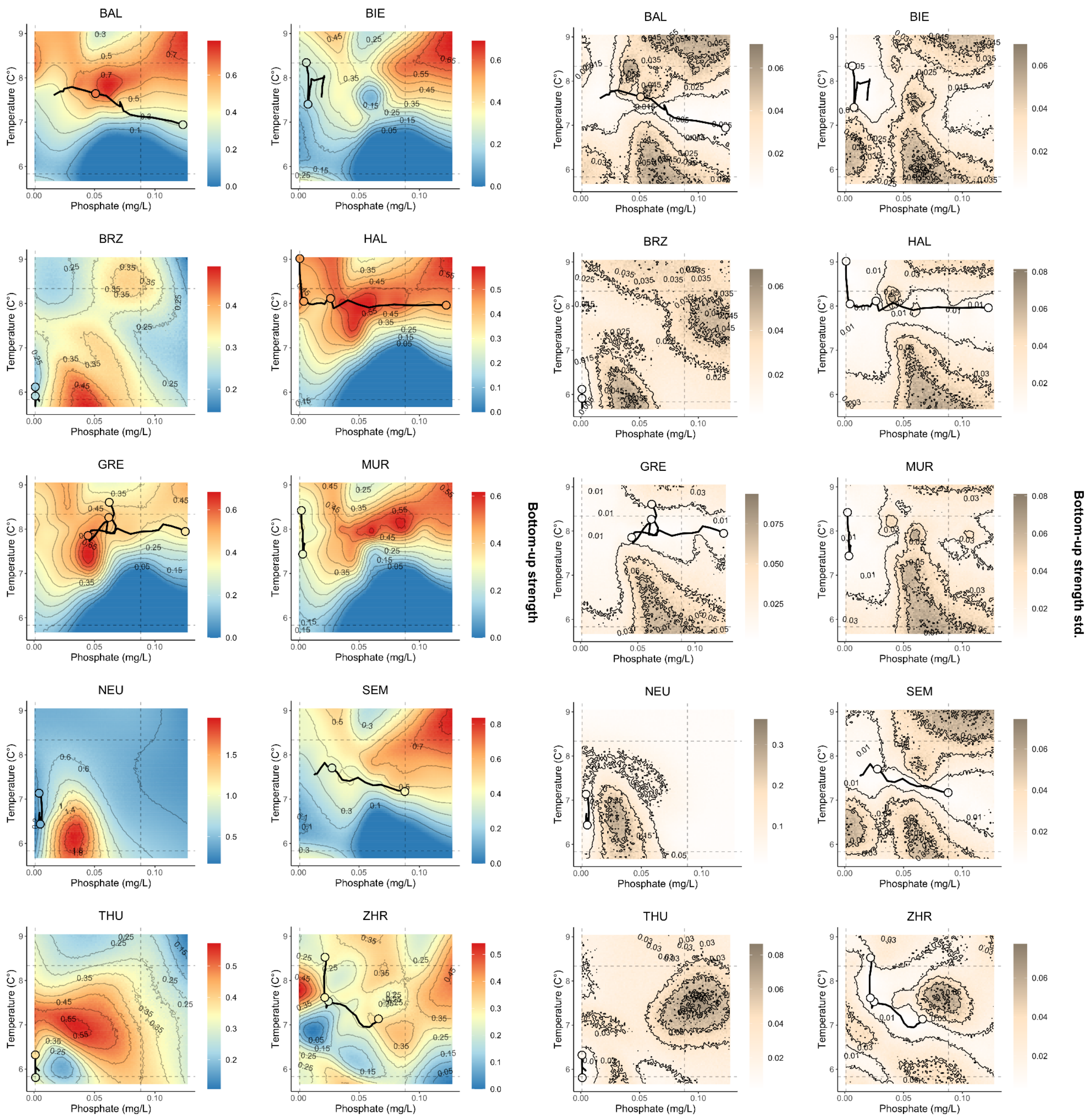
BAL
BIE
BRZ
HAL
GRE
MUR
NEU
SEM
THU
ZHR
Temperature (C°)
Phosphate (mg/L)
Bottom-up strength
Bottom-up strength std.

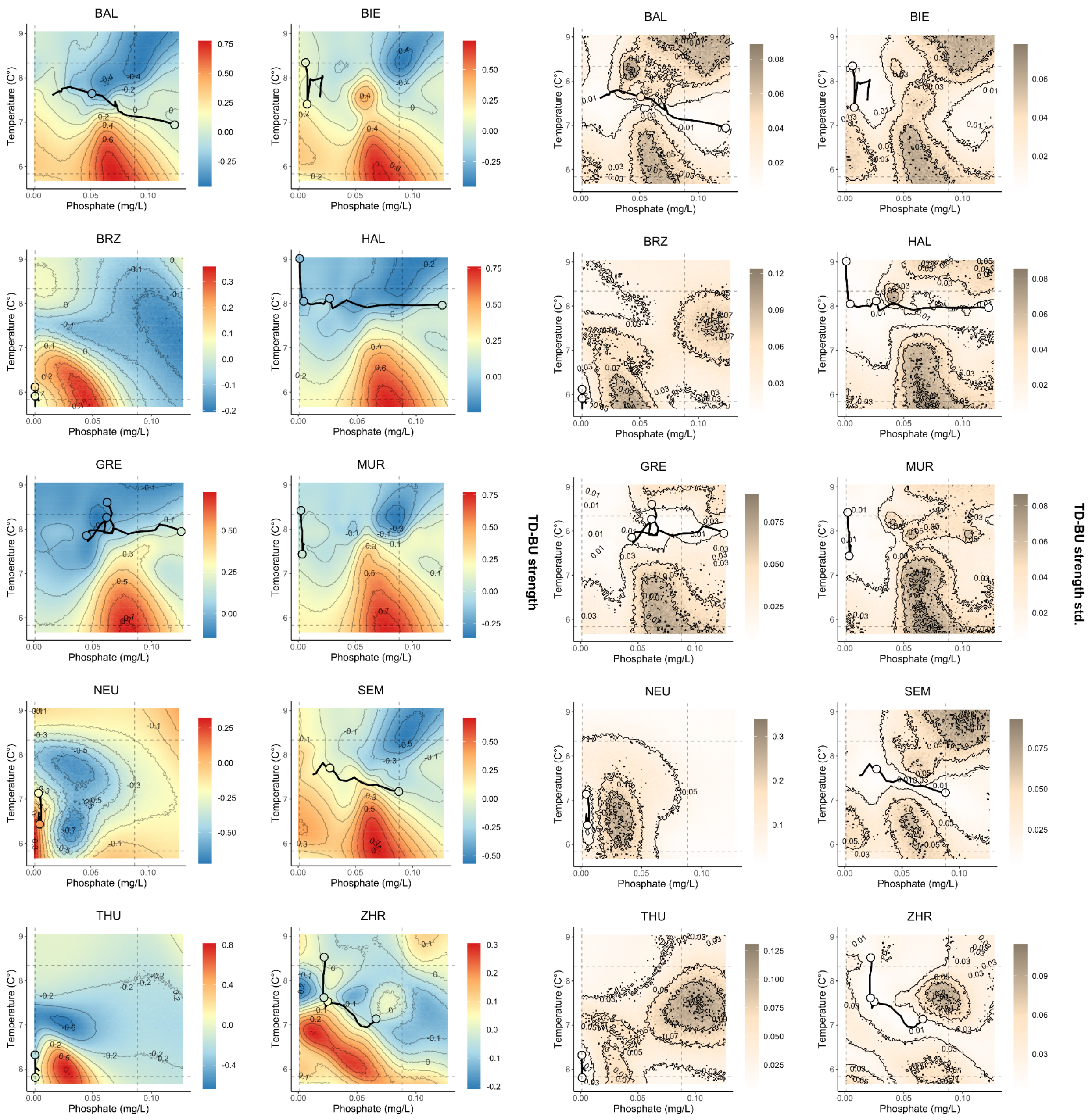

Fig. S5 S-map results for predicting network properties using water temperature and phosphate levels in the ten lakes. Combined effects of average water-column phosphate levels and temperature on realized connectance, average interactions strengths, and trophic controls: color-coded contour plots depict the s-map model inferred relationships and their confidence (standard deviation of prediction), which emerge from predicting network properties (z-axis) over varying levels of the chosen pair of explanatory variables (water temperature and phosphate), across the entire dataset while keeping lake depth and volume constant (**Methods**). Water temperature (y-axis), phosphate levels (x-axis), and

network properties (legends) ranged between the minimum and maximum observed values across all lakes. Dots depict the start/end of the re-oligotrophication and net-warming phase. Trajectories show the direction of time. The displayed year is the middle point of a 5-year time window.

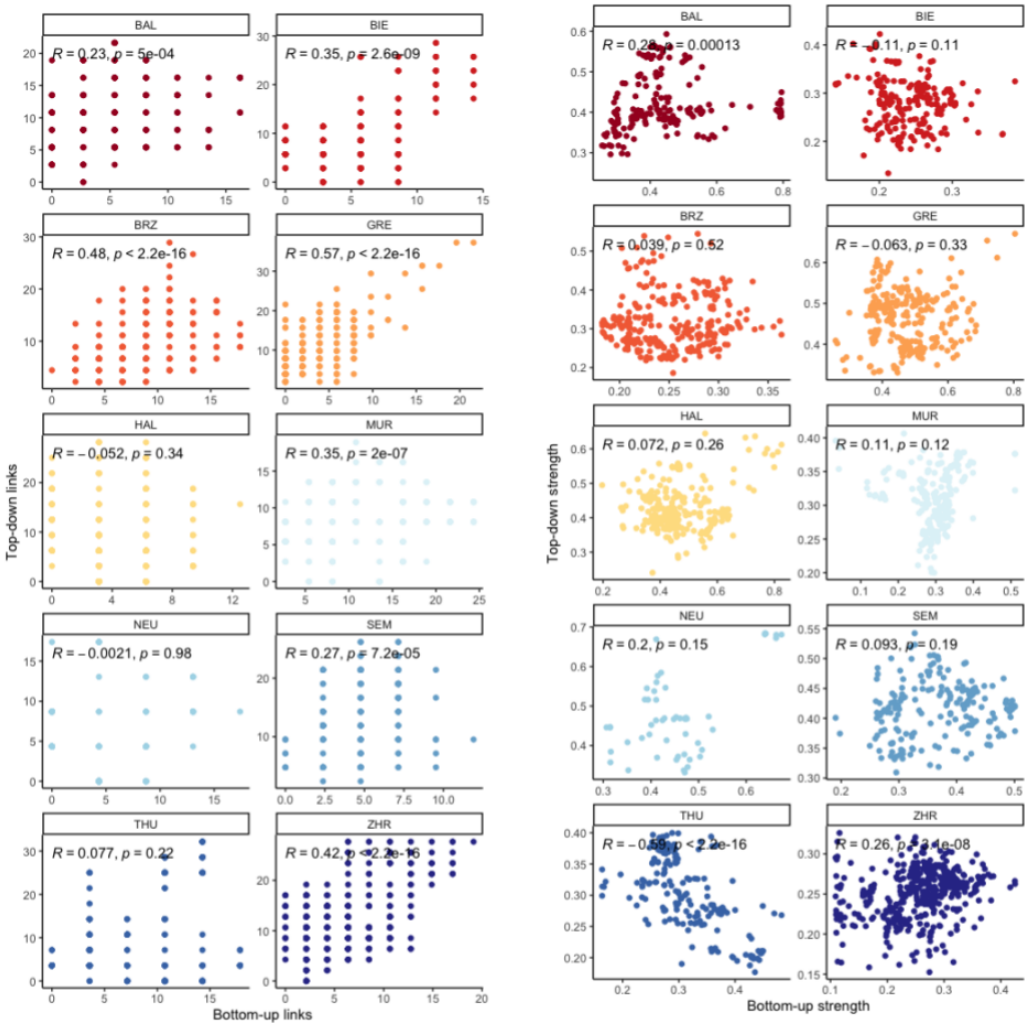

Fig. S6 Spearman-rank correlation between top-down and bottom-up control in the ten lakes. [a] **realized links** and [b] **strength**. Seven out of ten lakes display a positive significant correlation between the realized number of top-down and bottom-up links. Three out of ten lakes display a significant correlation between the strength of top-down and bottom-up links, whereas this correlation is positive in ZHR and BAL, it is negative in THU.

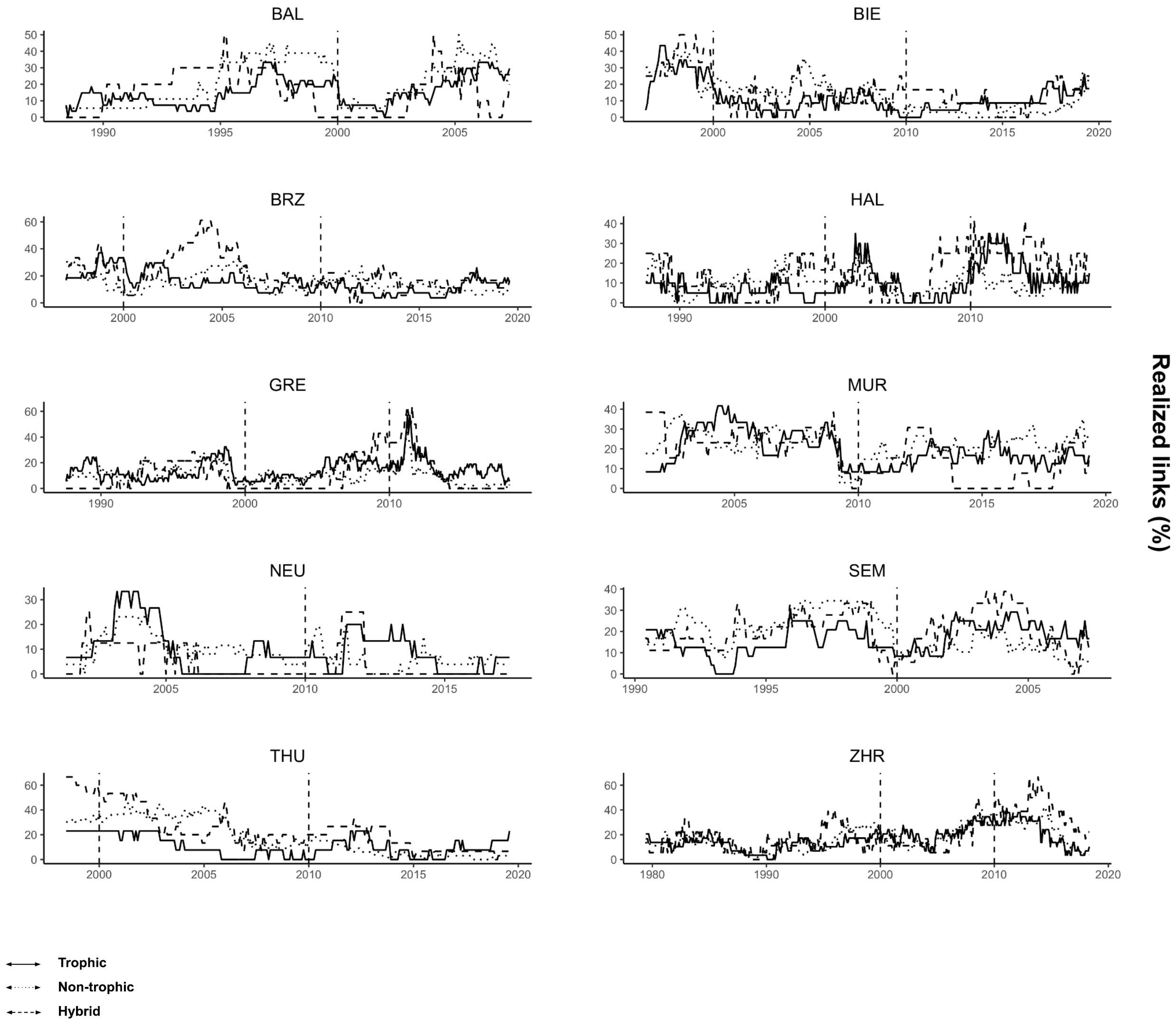
BAL
BIE
BRZ
HAL
GRE
MUR
NEU
SEM
THU
ZHR
Realized links (%)
Trophic
Non-trophic
Hybrid

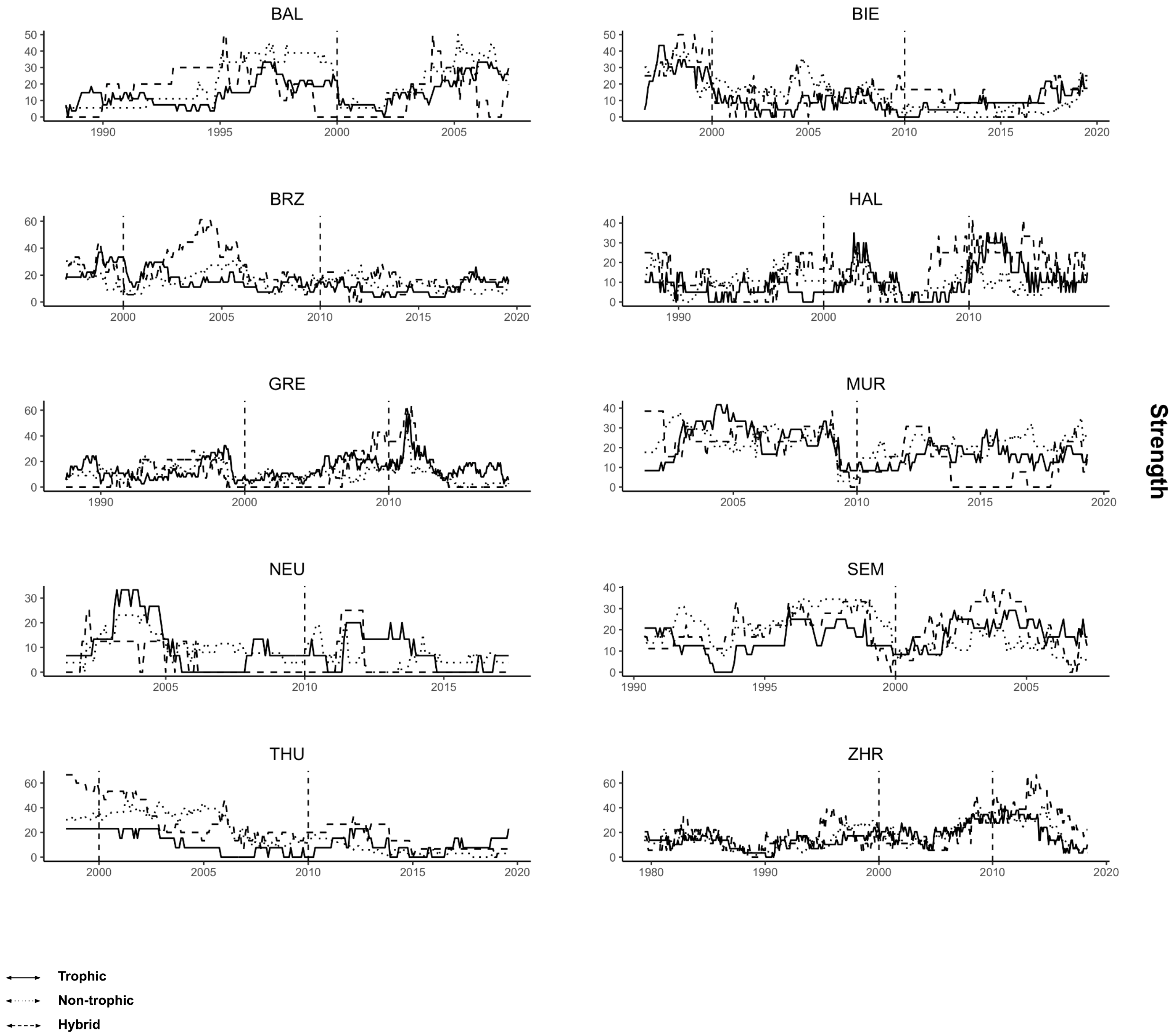

Fig. S7 Temporal changes in realized connectance (%) and strength of trophic, non-trophic and hybrid interactions. All lines are drawn based on the central point of a moving window of 60 months, used for causality detection via CCM.

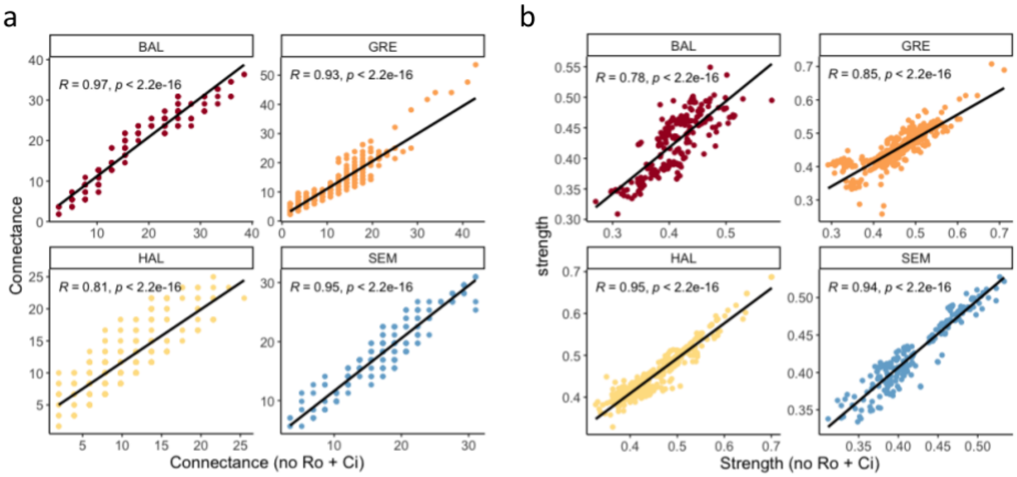

Fig. S8 Sensitivity analysis for connectance and interaction strength to the inclusion/exclusion of small grazers. To ensure that our results regarding connectance and interaction strength are comparable across lakes (some sampled small grazers, rotifers and ciliates, guilds Ro and Ci, while some others didn't), we ran the analyses for the lakes we had abundances for Ro and Ci with and without them. The major trends in connectance and interaction strength in the lakes BAL, GRE, HAL and SEM still hold when excluding ciliates and rotifers from the analysis.

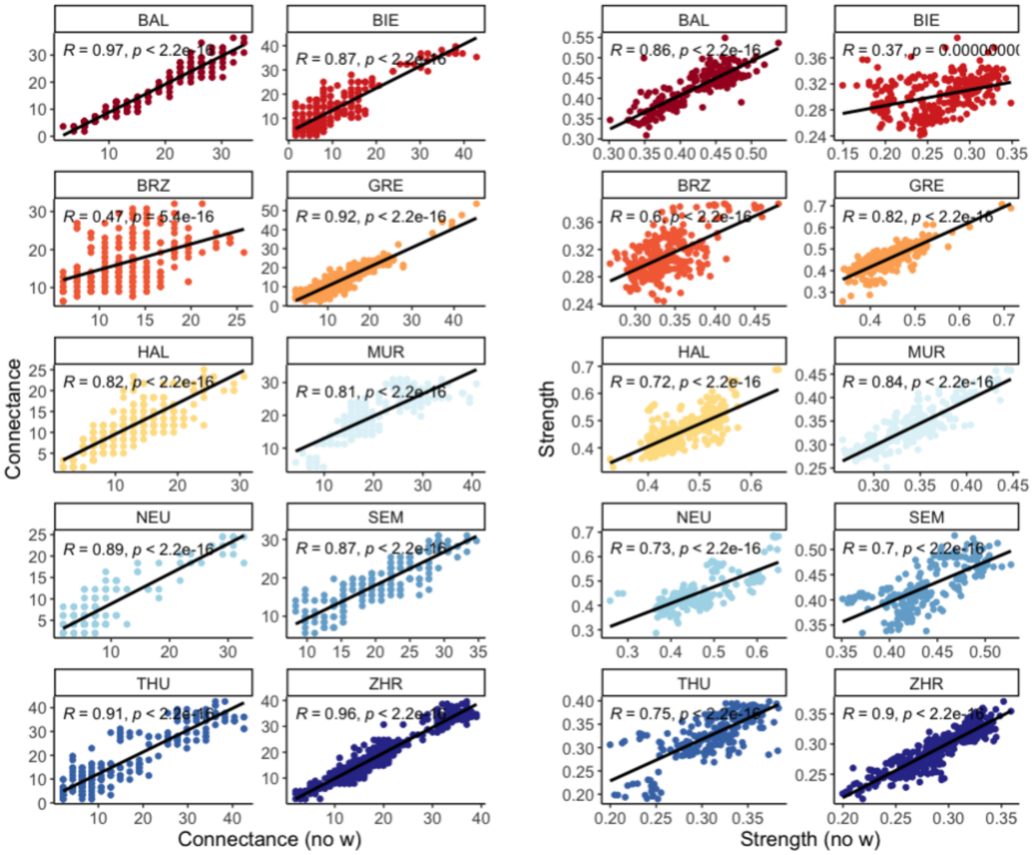

Fig. S9 Estimated connectance and interaction strength with and without winsorizing taxa abundance data. R and p-values are calculated using linear (Pearson's) correlation. Winsorizing taxa abundance data (raw data, before sorting into guilds) does not majorly alter [a] connectance nor [b] interaction strength.

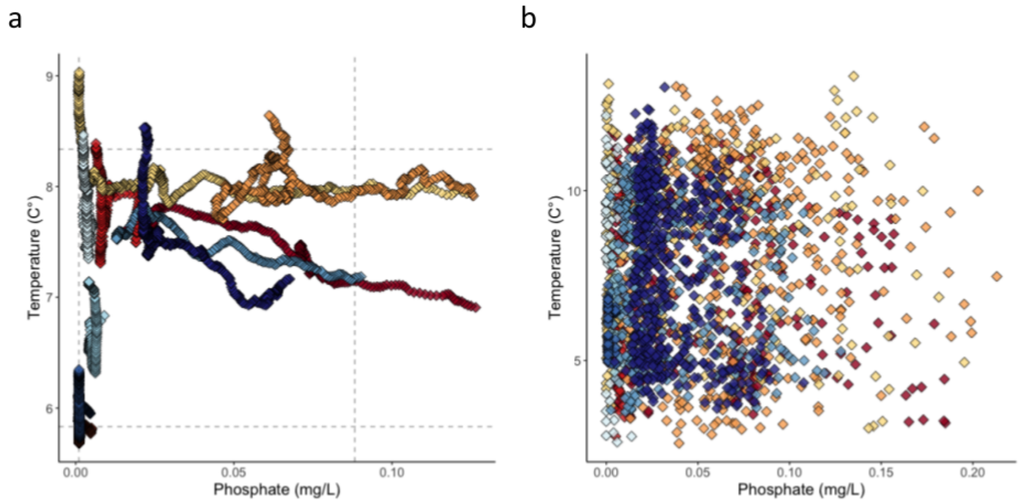

Fig. S10 Availability of temperature and phosphate concentration data for model predictions. [a] historical trajectories (values for water temperature and phosphate concentration) for each lake. Data were averaged within 60-month moving windows to match the time scale of the network properties. [b] Distribution of environmental data before averaging within 60-month moving windows.

Tab. S1 Lake metadata. Time points (n) include missing values as well. Taxa refer to the number of distinct taxonomic units sorted into guilds. (*) means variable sampling depths within this range over the years (**Methods**). Note: small zooplankton was not sampled in BIE, BRZ, MUR, NEU, THU, ZHR.

		Baldegg	Biel	Brienz	Greifen	Hallwil	Murten	Neuenburg	Sempach	Thun	Zurich
	abb.	BAL	BIE	BRZ	GRE	HAL	MUR	NEU	SEM	THU	ZHR
	volume (km3)	0.173	1.12	5.17	0.148	0.285	0.55	13.77	0.66	6.5	3.9
	Depth sampling site (m)	67	74	260	30	45	45	152	87	217	135
	Time span	1986-2009	1994-2022	1994-2022	1985-2020	1985-2020	1999-2021	1999-2019	1988-2009	1996-2022	1977-2020
	Missing values	6	6	2	24	19	4	18	6	2	5
	Time points (n)	288	337	331	430	427	276	252	264	314	528
Phytoplankton	Taxa (n)	274	332	182	334	300	303	195	263	215	293
	Records (n)	11385	20220	11099	20464	14390	12672	9718	11341	15488	35323
	Depth range	0-15 m	0-25 m*	0-40 m*	0-20 m	0-13 m	0-15 m*	0-40 m*	0-15 m	0-40 m*	0-135 m
	Winsorized data points (%)	4.2	3.3	3	3.6	3.9	4	3.5	4	2.9	2.7
Small zooplankton	Taxa (n)	53	NA	NA	58	57	NA	NA	49	NA	NA
	Records (n)	4348	NA	NA	6167	5244	NA	NA	3992	NA	NA
	Depth range	0-15 m	NA	NA	0-20 m	0-13 m	NA	NA	0-15 m	NA	NA
	Winsorized records (%)	2.4	NA	NA	2.1	2.2	NA	NA	2	NA	NA
Large zooplankton	Taxa (n)	61	77	49	50	56	50	48	58	53	98
	Records (n)	7939	9740	5086	6538	8948	5340	5591	7970	5908	13727
	Depth range	0-65 m	0-70 m	0-100 m	0-30 m	0-43 m	0-40 m	0-140 m	0-85 m	0_100 m	0-135 m
	Winsorized data points (%)	2.7	2.2	1.8	2.2	2.5	2.3	2.3	2.7	2	2.5
Chemistry	Depth range	0-65 m	0-74 m	0-260 m	0-30 m	0-45 m	0-45 m	0-152 m	0-85 m	0-217 m	0-135 m
	P-PO4 interpolated values	10	268	268	28	224	120	131	8	258	7
	T interpolated values	8	7	1	29	20	4	19	9	3	7
	P-PO4 level (ug/L) 1985-2020	0.056	NA	NA	0.073	0.048	NA	NA	0.039	NA	0.028
	ΔT(°C) 2005-2009 and 2016-2020	1.66	0.68	0.35	0.51	0.91	0.88	0.75	0.8	0.41	0.97

Tab. S2 S-map prediction accuracy. AL: All the lakes were included to make the predictions. LL: The lake of interest was left out to make the predictions. OL: Only the lake of interest was used to make the predictions. Rho is considered the predictive skill (Pearson's correlation between observations and observations). The degree of nonlinearity is measured by the maximum Δrho: the difference in Pearson's correlation between the best nonlinear model (when the locality parameter [Θ] in the S-maps is greater than 0) and the global linear model (when Θ = 0)

		Lake Baldegg			Lake Biel			Lake Brienz			Lake Greifen			Lake Hallwil		
	Approach	Rho (best theta)	Theta (best)	Rho (diff.)	Rho (best theta)	Theta (best)	Rho (diff.)	Rho (best theta)	Theta (best)	Rho (diff.)	Rho (best theta)	Theta (best)	Rho (diff.)	Rho (best theta)	Theta (best)	Rho (diff.)
Strength	AL	0.235	8	0.235	0.186	8	0.186	0.227	0	0.000	0.734	8	0.232	0.626	8	0.170
	LL	0.096	6	0.096	0.000	0	0.000	0.332	0	0.000	0.551	0	0.000	0.630	8	0.163
	OL	0.264	8	0.264	0.352	3	0.352	0.434	8	0.434	0.764	2	0.064	0.624	6	0.327
Connectance	AL	0.679	8	0.446	0.531	8	0.531	0.560	4	0.560	0.684	6	0.372	0.367	8	0.242
	LL	0.410	1	0.013	0.000	2	0.000	0.645	3	0.189	0.791	1.5	0.469	0.210	0	0.000
	OL	0.689	6	0.689	0.629	3	0.133	0.506	0.3	0.003	0.661	1.5	0.068	0.489	8	0.489
TD links	AL	0.198	0	0.000	0.138	8	0.138	0.398	6	0.398	0.668	6	0.668	0.505	8	0.218
	LL	0.275	0	0.000	0.000	0.75	0.000	0.587	8	0.587	0.162	6	0.162	0.400	0	0.000
	OL	0.433	8	0.342	0.354	3	0.191	0.395	0	0.000	0.656	1.5	0.073	0.642	8	0.490
BU links	AL	0.558	8	0.558	0.504	8	0.504	0.376	3	0.376	0.577	6	0.213	0.145	8	0.127
	LL	0.138	0	0.000	0.000	6	0.000	0.551	2	0.025	0.674	2	0.217	0.138	4	0.112
	OL	0.610	6	0.610	0.591	3	0.136	0.428	4	0.192	0.542	2	0.297	0.213	6	0.213
TD-BU links	AL	0.000	8	0.000	0.067	4	0.067	0.368	6	0.368	0.298	6	0.298	0.357	8	0.357
	LL	0.000	3	0.000	0.146	8	0.146	0.597	6	0.584	0.000	8	0.000	0.000	0	0.000
	OL	0.478	8	0.478	0.216	8	0.216	0.361	1.5	0.063	0.339	0	0.000	0.495	8	0.320
TD strength	AL	0.173	8	0.173	0.379	8	0.379	0.000	0	0.000	0.529	8	0.020	0.408	3	0.048
	LL	0.247	8	0.247	0.000	0	0.000	0.020	8	0.020	0.636	0	0.000	0.477	4	0.087
	OL	0.321	8	0.321	0.553	6	0.528	0.229	8	0.229	0.524	6	0.010	0.148	8	0.079
BU strength	AL	0.513	8	0.513	0.489	3	0.489	0.268	0.1	0.000	0.488	8	0.488	0.464	8	0.274
	LL	0.083	8	0.083	0.483	1	0.483	0.413	0	0.000	0.457	8	0.457	0.390	3	0.171
	OL	0.591	4	0.027	0.492	4	0.201	0.115	0	0.000	0.526	0.75	0.085	0.467	4	0.467
TD-BU strength	AL	0.180	8	0.180	0.318	8	0.318	0.000	8	0.000	0.551	6	0.551	0.323	8	0.323
	LL	0.376	0.1	0.016	0.134	3	0.134	0.000	8	0.000	0.674	8	0.674	0.310	1.5	0.310
	OL	0.637	8	0.161	0.459	8	0.214	0.100	8	0.100	0.592	0.3	0.004	0.379	8	0.379

Tab. S2: S-map prediction accuracy. (cont.)

		Lake Murten			Lake Neuchatel			Lake Sempach			Lake Thun			Lake Zurich		
	Approach	Rho (best theta)	Theta (best)	Rho (diff.)	Rho (best theta)	Theta (best)	Rho (diff.)	Rho (best theta)	Theta (best)	Rho (diff.)	Rho (best theta)	Theta (best)	Rho (diff.)	Rho (best theta)	Theta (best)	Rho (diff.)
Strength	AL	0.000	0	0.000	0.099	6	0.044	0.478	6	0.238	0.211	0	0.000	0.669	8	0.374
	LL	0.265	6	0.265	0.431	0	0.000	0.436	2	0.174	0.347	8	0.025	0.271	6	0.057
	OL	0.143	8	0.143	0.169	0	0.000	0.428	8	0.428	0.247	0	0.000	0.656	3	0.624
Connectance	AL	0.000	8	0.000	0.000	8	0.000	0.011	8	0.011	0.634	8	0.634	0.868	8	0.868
	LL	0.096	1	0.096	0.327	0.3	0.019	0.094	1.5	0.079	0.325	8	0.325	0.237	6	0.237
	OL	0.403	8	0.403	0.014	4	0.014	0.259	8	0.259	0.631	1	0.060	0.910	8	0.688
TD links	AL	0.000	8	0.000	0.000	0.75	0.000	0.142	0	0.000	0.760	6	0.760	0.762	8	0.534
	LL	0.000	0	0.000	0.162	8	0.162	0.226	0	0.000	0.710	8	0.710	0.381	6	0.381
	OL	0.081	8	0.081	0.000	0	0.000	0.103	8	0.103	0.775	2	0.072	0.777	6	0.430
BU links	AL	0.142	8	0.142	0.148	8	0.148	0.209	0	0.000	0.554	8	0.527	0.698	8	0.698
	LL	0.147	8	0.034	0.309	0.75	0.011	0.229	0.1	0.000	0.493	3	0.063	0.000	8	0.000
	OL	0.308	8	0.308	0.261	4	0.261	0.000	8	0.000	0.638	6	0.166	0.752	6	0.752
TD-BU links	AL	0.000	8	0.000	0.014	8	0.014	0.000	3	0.000	0.557	8	0.557	0.478	8	0.405
	LL	0.299	8	0.299	0.104	0	0.000	0.266	8	0.266	0.556	8	0.317	0.474	6	0.347
	OL	0.011	8	0.011	0.000	0.5	0.000	0.064	8	0.064	0.618	1.5	0.179	0.503	6	0.110
TD strength	AL	0.179	0	0.000	0.200	8	0.200	0.119	8	0.062	0.655	8	0.429	0.424	8	0.424
	LL	0.218	0	0.000	0.479	0	0.000	0.090	1	0.012	0.389	0	0.000	0.000	4	0.000
	OL	0.335	8	0.335	0.316	4	0.156	0.102	8	0.102	0.689	1.5	0.179	0.439	6	0.439
BU strength	AL	0.000	0	0.000	0.360	8	0.360	0.481	0.5	0.009	0.179	8	0.179	0.407	8	0.281
	LL	0.054	1.5	0.054	0.395	0	0.000	0.515	0.75	0.024	0.000	3	0.000	0.319	0	0.000
	OL	0.000	8	0.000	0.609	3	0.574	0.451	8	0.205	0.480	6	0.480	0.402	2	0.285
TD-BU strength	AL	0.000	2	0.000	0.000	8	0.000	0.541	8	0.541	0.542	8	0.542	0.294	8	0.294
	LL	0.144	0.03	0.000	0.530	0.5	0.067	0.141	0.1	0.017	0.301	0	0.000	0.086	2	0.086
	OL	0.000	8	0.000	0.229	8	0.229	0.599	6	0.506	0.718	6	0.641	0.292	2	0.118

Tab. S3 Best embedding dimension per network node and environmental variables in the ten lakes. The embedding dimension was estimated using simplex projection over E 2:15 (**Methods**).

	BAL	BIE	BRZ	GRE	HAL	MUR	NEU	SEM	THU	ZHR
Cy	4	13	15	9	9	11	10	13	15	14
Gr1	13	12	13	15	12	13	12	15	4	15
Gr2	8	15	15	11	5	14	5	11	11	12
Di1	3	13	12	12	15	11	8	12	7	11
Di2	12	14	5	6	11	13	6	13	12	15
Go1	12	15	15	5	15	3	9	8	15	15
Go2	5	11	15	3	15	14	7	13	14	15
Cr1	5	11	15	13	13	15	12	12	13	13
Cr2	8	13	11	12	12	11	12	15	3	14
Mi	15	15	15	12	11	15	7	14	15	15
Ci	12	NA	NA	13	6	NA	NA	6	NA	NA
Ro	4	NA	NA	14	7	NA	NA	3	NA	NA
He	11	13	14	14	12	15	11	14	12	15
Om	14	15	15	8	15	12	7	13	12	15
Pr	11	12	6	14	15	13	10	12	15	11
T	11	10	10	12	10	9	11	11	9	10
P	11	5	2	11	11	2	5	13	2	13

Methods (extended)

Data collection

Plankton abundance time series

Plankton samples were collected between 1977 and 2020 monthly across 10 Swiss lakes (**Fig. 1, Tab. S1**). Lake code names are BAL = Baldegg, BIE = Biel, BRZ = Brienz, GRE =Greifen, HAL = Hallwil, MUR = Murten, NEU = Neuenburg, SEM = Sempach, THU = Thun and ZHR = Zurich. In Lake Baldegg and Sempach, data from 2010 onwards were excluded from analyses due to irregular plankton sampling after phosphate levels stabilized (i.e., bi- or tri-monthly). Samples in all lakes have been collected at identical locations over the years and counted by the same taxonomists for each lake.

Phytoplankton and small zooplankton grazers (i.e., rotifers and ciliates) were sampled integrated over the water column in the photosynthetic zone using a Schröder sampler [1] or at discrete depths, where the lowest depth varied across lakes (**Tab. S1**). Sampling depth changed in BRZ from 0-20 m to 0-40 m in 2012, in BIE from 0-10 m to 0-15 m in 1999 and to 0-20 m in 2012, in MUR from 0-10 m to 0-15 m in 2012, in NEU from 0-20 m to 0-40 m in 2012; and in THU from 0-20 m to 0-40 m in 2012. Taxa abundances were converted to cells/L to compare across lakes. In Lake Zurich, the sampling method was changed in 2012 from discrete depth sampling (0, 1, 2.5, 5, 7.5, 10, 12.5, 15, 20, 30, 40, 60, 80, 100, 120, 130, 135 m) to integrated sampling (<20 m, 20-40 m and >40 m of the water column). To compare discrete with integrated samples, we multiplied each discrete sample by a conversion factor (obtained from a year of sampling where both methods were used simultaneously and biomass estimates between samplings were comparable) and aggregated them to match the corresponding integrated samples, e.g. multiplied discrete samples within 0 to 20 m by their corresponding factor and summed them up to match the integrated samples of < 20 m. Lake Biel, Baldegg, Murten, Neuenburg, Thun and Zurichsampling did not consider small grazers (ciliates and rotifers).

Large zooplankton was sampled using net-tows going from the bottom of the lake to the surface. Specific details about the lake sampling protocols can be found elsewhere[2,3]. Zooplankton densities were converted to individuals/m2 to compare across lakes. A full taxonomic list of species considered within this study can be found in an open-access data repository linked to this article (**link to ERIC server**). Plankton abundance data were winsorized, where values lying outside the 99% quantile were replaced by the highest values within the 99% quantile using the function **Winsorize** from the R package **DescTools** (**v**

0.99.43). This was done to reduce the power of large outliers without deleting data, since small typos can lead to large outliers in plankton counts. The main trends in connectance and interaction strength were robust to winsorizing (**Fig. S14**).

Water temperature and nutrient availability as environmental drivers

Chemical and physical parameters were measured monthly (occasionally bi- or tri-monthly) in the same locations where plankton samples were collected. Samples were taken from the surface to the lake's bottom at discrete depths. We focused on two main drivers of anthropogenic change in Swiss lakes, water temperature and freely available dissolved phosphate (PO_4)[4,5]. We used mean water temperature and phosphate concentration over the whole water column. Missing values were estimated using linear interpolation with **na_approx** from the R package **zoo (v 1.8-9)**. The approximated values ranged between 1 and 268 (**Tab. S1**). After re-oligotrophication, PO_4 levels remained constant and often below the detection limit in the lakes Biel, Brienz, Hallwil, Murten, Neuchatel and Thun. Sampling for nutrients in those lakes was changed to bi- or tri-monthly early, resulting in 120-268 approximated values.

Conceptual planktonic network

To understand processes at the network level and control for potential biases in taxa classification across lakes and over time, we aggregated plankton taxa abundances into a conceptual network based on taxonomic classification, body size, and feeding behavior[6]. This allowed us to overcome the limitations of a monitoring frequency lower than the generation time of the organisms (monthly sampling and noise in time series), and account for the intrinsic variability of species interactions while reducing the potential effects of taxonomic misclassification[7]. The dynamics of trophic guilds occur at the scale of months, as opposed to the dynamics of taxa which occur at the scale of days, and thus well represent seasonal and interannual network transitions[6,8].

Our conceptual network consisted of up to 15 nodes (guilds) across three trophic levels of the food web, containing large invertebrate predators, omnivores, large herbivore grazers, small grazers, mixotrophs, and primary producers. In Lake Biel, Brienz, Murten, Neuenburg and Zurich, we only had 13 guilds because of missing counts for rotifers and ciliates. We conducted a sensitivity analysis where we excluded rotifers (R) and ciliates (Ci) from Lake Baldegg, Greifen, Hallwil and Sempach data. Connectance and interaction strength were similar with and without rotifers (R) and ciliates (Ci) (**Fig. S13**). Because we could not differentiate between calanoid and cyclopoid nauplii nor their juvenile stage, and thus had

insufficient information on their feeding behavior, nauplii were excluded from our study. Small single cell cyanobacteria were excluded as well, since most taxa are below the size-detection limits of traditional microscopy.

The relationships (links) between nodes can be trophic (classic predator-prey relationship), non-trophic (i.e., mutualisms and competition) or hybrid, where guilds can have trophic or non-trophic relationships (i.e., mixotrophic dinoflagellates) (**Fig. 1c**). All links are bi-directional (in both directions), which means trophic and hybrid links can go up the network (BU), i.e., from a primary producer to a grazer, as well as down the network (top-down), i.e., from a grazer to a primary producer (**Fig. 1c**).

Data analysis

Chaos and nonlinear dynamics are ubiquitous in plankton communities, making linear statistical approaches unfit to study long-term changes in their network properties[9]. Linear statistical techniques are based on a correlation between two or more variables. In nonlinear systems, causality does not require correlation and correlation does not imply causation[10]. Nonlinear systems can exhibit state dependency, where the relationships between variables may change depending on the system's state. For example, large herbivore populations (**H**) may be affected by invertebrate predators (**C2**) only when their resource, phytoplankton (**P**), is scarce[11]. Equation-free approaches, such as empirical dynamic modeling (EDM), which can recover dynamics from empirical data, overcome this limitation and offer a promising non-parametric way to study nonlinear systems.

Empirical dynamic modeling (EDM) is rooted in state-space reconstruction, which does not assume any equations governing the system and recovers dynamics from empirical data. In EDM, a state space (i.e., an attractor) is reconstructed using time series belonging to the same dynamical system. An attractor is a description of rules that govern the system - without any a priori assumptions. For example, suppose the dynamics of large herbivores (**H**) are affected by phytoplankton (**P**) and invertebrate predators (**C2**). In that case, an attractor of the system's dynamics can be reconstructed by plotting the time series of **H**, **P** and **C2** along the x,y, and z axes. An educative animation on state-space reconstruction can be found here: https://deepeco.ucsd.edu/videos/#page-content.

In practice, we typically don't have knowledge of or data on every variable in a system. Takens' Theorem (1981), however, postulated that one can substitute any unknown or unobserved variables with a lag of a

single time series to reconstruct the system's attractor[12]. If two variables are from the same dynamical system, i.e. large herbivores (**H**) and phytoplankton (**P**), information about current phytoplankton populations will be encoded in past large herbivores' time series. Thus, if we have no information on phytoplankton (**P**) and invertebrate predators (**C2**), the system from above can be reconstructed using large herbivore abundance (**H**) at **t-τ** and **H** abundance at **t-2τ** respectively, where **τ** stands for time lag (e.g. 1 month).

To reconstruct the attractor of a system properly, one needs to know the ideal embedding. The ideal embedding is defined by the number of variables or lags used to build a system's attractor, each of them representing a coordinate axis; and provides information on the complexity of the system and the quality of data (i.e. highly resolved time series contain more information on the system and will thus result in a higher ideal embedding dimension). In our example above, the embedding dimension was set arbitrarily to three and a shadow attractor was reconstructed using large herbivore abundance (**H**) at **t**, **t-τ** and **t-2τ**. Ideally, we would define the best embedding first and then reconstruct the attractor using (**H(t), H(t-τ), H(t-2τ), …, H(t-(E)τ**), where **τ** is the desired lag (i.e. one month) and **E** the best embedding (see below for further explications).

Empirical dynamic modeling, can be used to determine the number of dimensions required to describe a system (best embedding)[13,14], quantify the non-linearity of time series[15–17], forecast future system states[13,18–20], infer causality between two variables[10], and to quantify how relationships (interactions) between variables change with changing system state[21]. We expanded the classical empirical dynamical framework by adding a temporal component to convergent cross-mapping (studying local correlations among observations and predictions within a moving window) and using the predictive skill rho (corrected for seasonality) as a proxy for how strongly a variable interacts and/or affects another variable. Below we briefly introduce the EDM concepts used in this paper and how we applied them to our data. Please refer to the cited references for proof of concepts and further information.

All analyses were performed using the **rEDM** package in R **(v-0.7.5)**. Up-to-date versions of rEDM can be found at https://github.com/SugiharaLab/rEDM. More information to perform EDM analyses can be found here: https://deepeco.ucsd.edu/resources/#page-content. "Empirical dynamic modeling for beginners" is another helpful resource, addressing basic applications with step-by-step example code [22].

Optimal Embedding Dimension

To reconstruct the dynamic attractor, one needs to know the ideal embedding dimension, i.e. the number of independent variables. The ideal embedding can be defined with simplex projection, a forecasting method relying on nearest neighbors [13]. Simplex projection uses only neighboring points in the state space of the predicted variable to make forecasts. The highest prediction skill (rho, Pearson's correlation ρ between the observed and the predicted values) indicates the optimal embedding dimension. If the right number of lags are used, the attractor reconstruction will map closely to that of the true underlying attractor of the system. If embeddings are insufficient, points corresponding to different system states will overlap in the reconstruction and thus hinder forecast accuracy.

Convergent Cross-Mapping

Convergent cross-mapping (CCM) is considered a "non-linear causality test" and assesses if one variable significantly affects another. As a consequence of Taken's theorem, univariate attractor reconstructions map to the original system and each other. By testing for mapping between two univariate attractor reconstructions, we can determine if two variables belong to the same system and thus share a causal relationship [10]. If large herbivores (**H**), phytoplankton (**P**) and invertebrate predators (**C2**) belong to the same system (are causally linked) and the best embedding is 3, the attractor (**H(t), H(t-τ), H(t-2τ)**) maps to the attractor (**H(t), P(t), C2(t)**) as well as (**P(t), P(t-τ), P(t-2τ)**) and (**C2(t), C2(t-τ), C2(t-2τ)**). In practice, this is done by testing how well a variable can be predicted using another variable's attractor reconstruction. We measure the forecasting skill rho (Pearson's correlation between predictions and observations), also called the "cross-mapping skill". For example, we may use the univariate reconstructed attractor (**P(t), P(t-τ), P(t-2τ)**) based on phytoplankton (**P**) to predict large herbivore abundance (**H**) at **t** or **t-τ**. Note that some causal relationships may be unidirectional, e.g., temperature will map to large herbivores (**H**) but not vice versa.

Convergence and surrogate time series are critical components of inferring causality between two variables using empirical data. Cross-mapping from one variable to another shall be "convergent", i.e., the predictive skill rho improves with library size (time-series length). Reconstructed univariate attractors become denser with more library points and thus, forecasts using nearest neighbors become better. The state space is reconstructed using different library lengths (number of data points) subsampled randomly from the time series to test for convergence. Seasonal co-occurrence patterns might obscure a causal relationship of one variable to another, which is especially true for plankton communities (i.e. PEG model [23]. Two variables with a seasonal cycle can have a high prediction skill, even if they don't share a causal link. Therefore, one can compare the forecasting skill rho of the original time series to rho estimated from

seasonal surrogates. Seasonal surrogate time series are created by randomly reshuffling the time series while keeping the seasonal signal (i.e., reshuffling among months).

Local cross-mapping skills (rho) describe changes in significant links over time and can be used to estimate time-varying (interaction) strength. Cross-mapping-skill is usually calculated over the whole time series. Local cross-mapping skills document changes in (plankton) networks and aid in overcoming a common trend. Instead of calculating Pearson's correlation between all observations and predictions, cross-mapping skills are estimated within a moving window. This results in a time series of cross-mapping skills, which can be used to derive time-dependent causal links when compared to seasonal surrogate time series. The local cross-mapping skill can be used as a proxy for the strength of the causal link when subtracting the seasonal component (i.e., $rho_{originalTS}$ - mean ($rho_{surrogateTS}$)). If this is done for a link between two network nodes, i.e., phytoplankton (P) and large herbivores (H), local cross-mapping skills can be used as a proxy for interaction strength between those two nodes.

Chaos and nonlinear dynamics are ubiquitous in plankton communities, making linear statistical approaches unfit to study long-term changes in their network properties[9]. In particular, nonlinear dynamics can obscure correlations between variables making causal links undetectable with classical statistical methods. Equation-free approaches, such as empirical dynamic modeling (EDM), which can recover dynamics from empirical data, overcome this limitation and offer a promising non-parametric way to study nonlinear systems (see http://tinyurl.com/EDM-intro for a brief video introduction). Empirical dynamic modeling, which is rooted in state-space reconstruction, can be used to determine the number of dimensions required to describe a system (best embedding)[13,14], quantify the non-linearity of time series[15–17], forecast future system states[13,18–20], infer causality between two variables[10], and to quantify how relationships (interactions) between variables change with changing system state[21]. A more in-depth description of EDM can be found in **Supplementary Information**. We expanded the classical EDM framework by adding a temporal component to convergent cross-mapping (studying local correlations among observations and predictions within a moving window)[10] and using the predictive skill rho (corrected for seasonality) as a proxy for how strongly a network node is affected and/or affects another node. Moreover, we used S-maps to explore the interactive effects of water temperature and nutrient levels on network properties[15].

Reconstructing a time-varying causal network using convergent cross-mapping

Consider two variables, V1 (e.g., phytoplankton) and V2 (e.g., large herbivores or temperature). We want to know whether and how strongly V1 is impacted by V2; i.e., V1 ← V2. This is determined by measuring how much V2 has impacted the dynamics (time series) of V1 – how much information about V2 has been imprinted in the time series of V1. This information allows one to use V1 to estimate the states of the driver V2, a process known as cross-mapping between variables[10]. The stronger the signature (causal impact in the affected variable) the better the cross-map estimate. To do this in the R package rEDM, we would call V1 xmap V2 where again the direction of effect we are testing is V1 ← V2. Note that the time series require added placeholders for missing values to ensure having evenly spaced monthly data. Since the time series used are on different scales (e.g.; temperature measurements and abundance data), we rescale them using the function **scale** in the R package **base** (**v-4.1.0**).

Embedding dimension. We use **simplex** from the R package **rEDM (v-0.7.5)** to define the best embedding dimension for V1 using simplex projection (**Supplementary Information**). The embedding dimension was run over E = 2:15. Time lag and prediction horizon were set to 1 month. The number of nearest neighbors used to make predictions are set to E+1. Forecasting was done using leave-one-out cross-validation and the best embedding was selected based on maximizing the forecasting skill rho (**Tab. S3**).

Convergence test. We tested the convergence of V1 xmap V2 by comparing the predictive power of using 20% and 50% of the data, respectively. This was done with 100 consecutive random subsets of the time series. The ideal embedding dimension was defined for V1 based on forecasting with simplex-projection (see above and **Tab. S3**), while the time lag tp was kept at 0. Convergent cross-mapping was run with the function **ccm** from the R package **rEDM** (**v-0.7.5**). Convergence was considered true if $rho_{50\%} > rho_{20\%}$ for the 100 subsets, determined by a one-sided t-test (95% quantile).

Local cross-mapping-skill (rho). If the convergence test was significant, we performed CCM between V1 and V2 this time using the maximum library (whole time series) and tp = -1. Using the predictions from the CCM-output, we calculated local rho's, i.e. the correlation between observation of V2 and predictions of V2 (using V1's attractor) within moving windows (n=60 months, sliding 1 month forward at a time). This resulted in a time series of rho's (forecast skills).

Seasonal surrogates. The local rho's were then compared to rho's from 100 random seasonal surrogate time series for each time window (time point t_x). We considered the link V1 ← V2 at time point t_x as significant if 95% of the times $rho_{originalTS} > rho_{surrogateTS}$. If the link was significant, we estimate the

strength of V1 ← V2 at t_x by removing the seasonal component from the local $rho_{originalTS}$, i.e., $rho_{originalTS}$ - mean ($rho_{surrogateTS}$), the average local rho of the 100 surrogate time series. Negative rho's were always set to 0.

Network links. To calculate network connectance, we summed all causal links (passed the surrogate test) per lake and date (month) and divided them by the total possible links for this network (based on the conceptual network in **Fig. 1c** and convergence test). We obtained connectance (%), the number of connected nodes, for this time point and a time series of connectance per lake (**Fig. 2a**).

Taxa interaction strength. We calculate the mean strength of links across nodes per date and lake. This resulted in average link strength for this time point and a time series of average link strength per lake (**Fig. 2b**). Taxa interaction strength over time and across lakes (**Fig. ED6**) was calculated by estimating the average strength of each link and multiplying it by its prevalence over time (per lake), i.e., corrected the strength for how often it occurred in the time series, and then average across lakes.

Environment effect on guilds. To get at the strength of water temperature and phosphate effects over time on each guild's abundance (**Fig. ED6**), we calculated the local cross-mapping-skill rho and compared it to a value obtained by a seasonal null model. We averaged the strength of water temperature and phosphate effects for each node and multiplied by its prevalence over time and then averaged across lakes (analogous to calculating interaction strengths between guilds over time across lakes).

Feedback between temperature and nutrients. To test for a causal relationship (feedback) between water temperature and phosphate concentration (**Fig. ED2**), we used CCM on the whole time series and performed a convergence test (n=100) and seasonal surrogate test (n=100). If both the convergence test ($rho_{50\%}$ > $rho_{20\%}$) and seasonal surrogate test (>95% of times $rho_{originalTS}$ > $rho_{surrogateTS}$) passed, we considered an effect as significant (lake displayed as points in **Fig. ED2**). To get a robust estimation of the effect's magnitude (i.e., filter out single episodic events and diminish the power of outliers), we multiplied the strength of the effect at each time point by its prevalence over time (per lake), i.e., we corrected the strength of the causal effect by how often a significant effect occurred in the time series. The resulting value was plotted on the y-axis in **Fig. ED2.**

Trophic controls. We summed up all causal links going up (bottom-up) and down (top-down) the food web (i.e., trophic and hybrid links) per time point and lake and divided them by all the total possible bottom-up or top-down links for this network (**Fig. 1c**). Moreover, we averaged the strength of all

significant bottom-up and top-down links per time point and lake. Then we calculated the difference between realized top-down and bottom-up links (i.e., top-down connectance - bottom-up connectance) and top-down and bottom-up strength (i.e., top-down link strength - bottom-up link strength). This resulted in a time series of changes in trophic controls over time, whereas a value > 0 indicated top-down and < 0 bottom-up control (**Fig. 4a-b**). If there were no significant bottom-up and/or top-down links at a given time point, connectance was set to 0 and strength to NA (unknown).

Interaction types. We summed up all trophic, non-trophic and hybrid links (according to **Fig. 1c**) per time point and lake and divide them by all total possible links per interaction type. Then we averaged connectance and interaction strength for trophic, non-trophic and hybrid links per time point across lakes. This resulted in a time series of connectance (%) and strength of trophic, non-trophic and hybrid links (**Fig. S7**). We compared connectance (%) and strength of interaction types using a Wilcoxon test, a non-parametric method for testing if samples originate from the same distribution (**Fig. 5**).

Scenario exploration using multivariate S-maps

We used multivariate S-maps to model network properties and extract their relationship with phosphate levels and water temperature. S-maps compute a unique locally weighted linear regression to make a forecast at each point in time when closer points on the attractor are given a higher weight. The strength of weighting is controlled by the parameter theta and indicates the degree of non-linearity and state dependency. Each regression provides a set of coefficients that define relationships (dynamics) between variables at each unique state. These coefficients were used to estimate (predict) each network property at varying levels of temperature and phosphate (**Fig. 2, 4 c-d, Fig. S4-5**). To account for important differences in the morphometry of lakes, which influence these ecosystems' responses to changes in nutrient inputs and warming, we included depth at the sampling site and lake total water volume in the S-map models[25]. Confidence in the predictions can be influenced by the parameter space covered by the lake time series, e.g., less confidence in the predictions for combinations of high water temperature and phosphate levels (**Fig. S10**).

We ran the s-map models using **rEDM (v-0.7.5)** and the function **block_lnlp** for 100 random subsets, using 50% of the data and averaged (mean) the predictions. The variance was calculated by estimating the standard deviation among the 100 predictions. Environmental drivers were smoothed within 60-month moving windows to match the temporal scale of modeled network properties. We chose 100 values for temperature and phosphate levels (each), ranging from the minimum to the maximum values observed

across all lakes. This resulted in a grid of 10000 model predictions (**Fig. 2, 4 c-d, Fig. S4-5**). Methods within the function were set to "s-map" and the exclusion radius to 12 to avoid the high temporal autocorrelation caused by the moving windows. Theta was selected to maximize predictive skill rho when varied over a list of values (0, 0.0001, 0.0003, 0.001, 0.003, 0.01, 0.03, 0.1, 0.3, 0.5, 0.75, 1.0, 1.5, 2, 3, 4, 6, and 8) and tp set to 0.

Methods references

1. Mieleitner, J. & Reichert, P. Modelling functional groups of phytoplankton in three lakes of different trophic state. *Ecol. Modell.* **211**, 279–291 (2008).
2. Pomati, F., Matthews, B., Jokela, J., Schildknecht, A. & Ibelings, B. W. Effects of re-oligotrophication and climate warming on plankton richness and community stability in a deep mesotrophic lake. *Oikos* **121**, 1317–1327 (2012).
3. Pomati, F., Shurin, J. B., Andersen, K. H., Tellenbach, C. & Barton, A. D. Interacting Temperature, Nutrients and Zooplankton Grazing Control Phytoplankton Size-Abundance Relationships in Eight Swiss Lakes. *Front. Microbiol.* **10**, 3155 (2019).
4. Monchamp, M.-E. *et al.* Homogenization of lake cyanobacterial communities over a century of climate change and eutrophication. *Nat Ecol Evol* **2**, 317–324 (2018).
5. Monchamp, M.-E., Spaak, P. & Pomati, F. High dispersal levels and lake warming are emergent drivers of cyanobacterial community assembly in peri-Alpine lakes. *Sci. Rep.* **9**, 7366 (2019).
6. Boit, A., Martinez, N. D., Williams, R. J. & Gaedke, U. Mechanistic theory and modelling of complex food-web dynamics in Lake Constance. *Ecol. Lett.* **15**, 594–602 (2012).
7. Pomati, F. *et al.* Challenges and prospects for interpreting long-term phytoplankton diversity changes in Lake Zurich (Switzerland). *Freshwater Biology* vol. 60 1052–1059 Preprint at https://doi.org/10.1111/fwb.12416 (2015).
8. Ehrlich, E. & Gaedke, U. Coupled changes in traits and biomasses cascading through a tritrophic plankton food web. *Limnol. Oceanogr.* **65**, 2502–2514 (2020).

9. Benincà, E. *et al.* Chaos in a long-term experiment with a plankton community. *Nature* **451**, 822–825 (2008).
10. Sugihara, G. *et al.* Detecting causality in complex ecosystems. *Science* **338**, 496–500 (2012).
11. van Someren Gréve, H., Kiørboe, T. & Almeda, R. Bottom-up behaviourally mediated trophic cascades in plankton food webs. *Proc. Biol. Sci.* **286**, 20181664 (2019).
12. Takens, F. Detecting strange attractors in turbulence. in *Dynamical Systems and Turbulence, Warwick 1980* 366–381 (Springer Berlin Heidelberg, 1981).
13. Sugihara, G. & May, R. M. Nonlinear forecasting as a way of distinguishing chaos from measurement error in time series. *Nature* **344**, 734–741 (1990).
14. Hsieh, C.-H., Glaser, S. M., Lucas, A. J. & Sugihara, G. Distinguishing random environmental fluctuations from ecological catastrophes for the North Pacific Ocean. *Nature* **435**, 336–340 (2005).
15. Sugihara, G., Grenfell, B. T., May, R. M. & Tong, H. Nonlinear forecasting for the classification of natural time series. *Philosophical Transactions of the Royal Society of London. Series A: Physical and Engineering Sciences* **348**, 477–495 (1994).
16. Anderson, C. N. K. *et al.* Why fishing magnifies fluctuations in fish abundance. *Nature* **452**, 835–839 (2008).
17. Sugihara, G. *et al.* Are exploited fish populations stable? *Proceedings of the National Academy of Sciences of the United States of America* vol. 108 E1224–5; author reply E1226 (2011).
18. Ye, H. & Sugihara, G. Information leverage in interconnected ecosystems: Overcoming the curse of dimensionality. *Science* **353**, 922–925 (2016).
19. Ye, H. *et al.* Equation-free mechanistic ecosystem forecasting using empirical dynamic modeling. *Proc. Natl. Acad. Sci. U. S. A.* **112**, E1569–76 (2015).
20. Dixon, P. A., Milicich, M. J. & Sugihara, G. Episodic fluctuations in larval supply. *Science* **283**, 1528–1530 (1999).
21. Deyle, E. R., May, R. M., Munch, S. B. & Sugihara, G. Tracking and forecasting ecosystem interactions in real time. *Proc. Biol. Sci.* **283**, (2016).

22. Chang, C.-W., Ushio, M. & Hsieh, C.-H. Empirical dynamic modeling for beginners. *Ecol. Res.* **32**, 785–796 (2017).

23. Sommer, U. *et al.* Beyond the Plankton Ecology Group (PEG) Model: Mechanisms Driving Plankton Succession. (2012) doi:10.1146/annurev-ecolsys-110411-160251.
