# Supplementary material for: Disruption of ecological networks in lakes by climate change and nutrient fluctuations
Source: Nat Clim Chang. 2023 Mar 23;13(4):389–96. doi: 10.1038/s41558-023-01615-6 (PMC10079529; doi:10.1038/s41558-023-01615-6)
Supplement: Supplementary file 2 — Reporting Summary [file 41558_2023_1615_MOESM2_ESM.pdf]

nature research

Corresponding author(s): Ewa Merz

Last updated by author(s): Jan 23, 2023

Reporting Summary

Nature Research wishes to improve the reproducibility of the work that we publish. This form provides structure for consistency and transparency in reporting. For further information on Nature Research policies, see our Editorial Policies and the Editorial Policy Checklist.

Statistics

For all statistical analyses, confirm that the following items are present in the figure legend, table legend, main text, or Methods section.

n/a	Confirmed	
☐	☒	The exact sample size (n) for each experimental group/condition, given as a discrete number and unit of measurement
☐	☒	A statement on whether measurements were taken from distinct samples or whether the same sample was measured repeatedly
☐	☒	The statistical test(s) used AND whether they are one- or two-sided<br>*Only common tests should be described solely by name; describe more complex techniques in the Methods section.*
☐	☒	A description of all covariates tested
☐	☒	A description of any assumptions or corrections, such as tests of normality and adjustment for multiple comparisons
☐	☒	A full description of the statistical parameters including central tendency (e.g. means) or other basic estimates (e.g. regression coefficient) AND variation (e.g. standard deviation) or associated estimates of uncertainty (e.g. confidence intervals)
☐	☒	For null hypothesis testing, the test statistic (e.g. F, t, r) with confidence intervals, effect sizes, degrees of freedom and P value noted<br>*Give P values as exact values whenever suitable.*
☒	☐	For Bayesian analysis, information on the choice of priors and Markov chain Monte Carlo settings
☒	☐	For hierarchical and complex designs, identification of the appropriate level for tests and full reporting of outcomes
☐	☒	Estimates of effect sizes (e.g. Cohen's d, Pearson's r), indicating how they were calculated

Our web collection on statistics for biologists contains articles on many of the points above.

Software and code

Policy information about availability of computer code

Data collection	R-Studio (1.4.1717)
Data analysis	R-Studio (1.4.1717)

For manuscripts utilizing custom algorithms or software that are central to the research but not yet described in published literature, software must be made available to editors and reviewers. We strongly encourage code deposition in a community repository (e.g. GitHub). See the Nature Research guidelines for submitting code & software for further information.

Data

Policy information about availability of data

All manuscripts must include a data availability statement. This statement should provide the following information, where applicable:

- Accession codes, unique identifiers, or web links for publicly available datasets
- A list of figures that have associated raw data
- A description of any restrictions on data availability

The data and code supporting the results are publicly available on Eric (https://doi.org/10.25678/0007VX).

Field-specific reporting

Please select the one below that is the best fit for your research. If you are not sure, read the appropriate sections before making your selection.

☐ Life sciences ☐ Behavioural & social sciences ☒ Ecological, evolutionary & environmental sciences

For a reference copy of the document with all sections, see nature.com/documents/nr-reporting-summary-flat.pdf

Ecological, evolutionary & environmental sciences study design

All studies must disclose on these points even when the disclosure is negative.

Study description	We study how climate change (rise in water temperature) and nutrient fluctuations (decrease in phosphate levels) interact to affect plankton networks in five Swiss lakes.
Research sample	zoo- and phytoplankton abundances
Sampling strategy	Samples were taken with a Schröder sampler (phytoplankton, small zooplankton) and net-tows (zooplankton). Two very well established methods for plankton sampling.
Data collection	Samples in all lakes have been collected at identical locations over the years and were counted by the same group of taxonomists. Phytoplankton and small zooplankton grazers (i.e., rotifers and ciliates) were sampled integrated over the water column using a Schröder sampler or at discrete depths, where the lowest depth varied across lakes. Taxa abundances were converted to cells/L to compare across lakes. In Zurichsee, the sampling method was changed in 2012 from discrete depth sampling (0, 1, 2.5, 5, 7.5, 10, 12.5, 15, 20, 30, 40, 60, 80, 100, 120, 130, 135 m) to integrated sampling (<20 m, 20-40 m and >40 m of the water column). To compare discrete with integrated samples, we multiplied each discrete sample by a conversion factor and aggregated them to match the corresponding integrated samples, e.g. multiplied discrete samples within 0 to 20 m by their corresponding factor and summed them up to match the integrated samples of < 20 m. Large zooplankton was sampled using net-tows going from the bottom of the lake to the surface. Details about the lake sampling protocols can be found elsewhere. Densities were converted to individuals/m2 to compare across lakes. Chemical and physical parameters were measured monthly (occasionally bi-monthly) in the same locations where plankton samples were collected. Samples were done from the surface to the lake's bottom at discrete depths. We focused on two main drivers of anthropogenic change in Swiss lakes, water temperature and freely available dissolved phosphate (PO4)
Timing and spatial scale	Plankton samples were collected between 1977 and 2020 monthly (occasionally bi-monthly) across 10 Swiss lakes
Data exclusions	If guilds (= network nodes) do not meet a 50% abundance criteria, i.e., present (values greater than 0) at least half of the time during the studied period, they were excluded from the analysis. All nodes were always present, so none of the guilds had to be excluded.
Reproducibility	no experiment was done
Randomization	no experiment was done
Blinding	no experiment was done

Did the study involve field work? ☒ Yes ☐ No

Field work, collection and transport

Field conditions	field conditions varied
Location	sampling was done in the middle of the lake, close to it's deepest part
Access & import/export	access by motorboat
Disturbance	no major disturbance was caused by the study

Reporting for specific materials, systems and methods

We require information from authors about some types of materials, experimental systems and methods used in many studies. Here, indicate whether each material, system or method listed is relevant to your study. If you are not sure if a list item applies to your research, read the appropriate section before selecting a response.

Materials & experimental systems

n/a	Involved in the study	
☒	☐	Antibodies
☒	☐	Eukaryotic cell lines
☒	☐	Palaeontology and archaeology
☐	☒	Animals and other organisms
☒	☐	Human research participants
☒	☐	Clinical data
☒	☐	Dual use research of concern

Methods

n/a	Involved in the study	
☒	☐	ChIP-seq
☒	☐	Flow cytometry
☒	☐	MRI-based neuroimaging

Animals and other organisms

Policy information about studies involving animals; ARRIVE guidelines recommended for reporting animal research

Laboratory animals	no laboratory animals were used
Wild animals	plankton samples fixed in ethanol and/or lugol for identification purposes
Field-collected samples	no field collected samples were used in experiments
Ethics oversight	no ethical approval or guidance was required

Note that full information on the approval of the study protocol must also be provided in the manuscript.
